# Microautophagy Mediates Vacuolar Delivery of Storage Proteins in Maize Aleurone Cells

**DOI:** 10.3389/fpls.2022.833612

**Published:** 2022-02-18

**Authors:** Xinxin Ding, Xiaoguo Zhang, Julio Paez-Valencia, Fionn McLoughlin, Francisca C. Reyes, Kengo Morohashi, Erich Grotewold, Richard D. Vierstra, Marisa S. Otegui

**Affiliations:** ^1^Department of Botany, University of Wisconsin-Madison, Madison, WI, United States; ^2^Center for Quantitative Cell Imaging, University of Wisconsin-Madison, Madison, WI, United States; ^3^Department of Biology, Washington University in St. Louis, St. Louis, MO, United States; ^4^Department of Biochemistry and Molecular Biology, Michigan State University, East Lansing, MI, United States

**Keywords:** endosperm, EUL lectin, phospholipase-D, tonoplast proteome, RNA-seq

## Abstract

The molecular machinery orchestrating microautophagy, whereby eukaryotic cells sequester autophagic cargo by direct invagination of the vacuolar/lysosomal membrane, is still largely unknown, especially in plants. Here, we demonstrate microautophagy of storage proteins in the maize aleurone cells of the endosperm and analyzed proteins with potential regulatory roles in this process. Within the cereal endosperm, starchy endosperm cells accumulate storage proteins (mostly prolamins) and starch whereas the peripheral aleurone cells store oils, storage proteins, and specialized metabolites. Although both cell types synthesize prolamins, they employ different pathways for their subcellular trafficking. Starchy endosperm cells accumulate prolamins in protein bodies within the endoplasmic reticulum (ER), whereas aleurone cells deliver prolamins to vacuoles *via* an autophagic mechanism, which we show is by direct association of ER prolamin bodies with the tonoplast followed by engulfment *via* microautophagy. To identify candidate proteins regulating this process, we performed RNA-seq transcriptomic comparisons of aleurone and starchy endosperm tissues during seed development and proteomic analysis on tonoplast-enriched fractions of aleurone cells. From these datasets, we identified 10 candidate proteins with potential roles in membrane modification and/or microautophagy, including phospholipase-Dα5 and a possible EUL-like lectin. We found that both proteins increased the frequency of tonoplast invaginations when overexpressed in *Arabidopsis* leaf protoplasts and are highly enriched at the tonoplast surface surrounding ER protein bodies in maize aleurone cells, thus supporting their potential connections to microautophagy. Collectively, this candidate list now provides useful tools to study microautophagy in plants.

## Introduction

The embryo and endosperm within seeds derive from a double fertilization event. Afterward, they follow very different developmental programs, with the embryo becoming the next generation and the endosperm accumulating storage compounds which are then consumed by the embryo to support development and seed germination. The maize endosperm consists of four main cell types with unique functions: the starchy endosperm cells, which constitute the bulk of the endosperm; the peripheral aleurone cells; the basal endosperm transfer layer; and the specialized cells that surround the embryo (Olsen, 2004).

The starchy endosperm and aleurone layer are the main sites for nutrient storage within maize seeds. Starchy endosperm cells undergo programmed cell death during development (Young and Gallie, 2000; Sabelli et al., 2013), whereas the aleurone single-cell layer acquires desiccation tolerance and remains alive at seed maturity (Hoecker et al., 1995). The starchy endosperm represents approximately 75% of seed weight and accumulates large amounts of starch granules and storage proteins that are mostly comprised of alcohol-soluble prolamins (called zeins in maize). During germination and in response to gibberellic acid synthesized by the embryo, aleurone cells secrete enzymes that help degrade starch and proteins with the resulting sugars and amino acids from the starchy endosperm then mobilized to nourish the embryo (Becraft and Yi, 2011). Aleurone cells also store nutrients, including storage proteins, lipids, and over 70% of the minerals found within the endosperm (Stewart et al., 1988; Becraft, 2011; Iwai et al., 2012).

Although, both aleurone and starchy endosperm cells accumulate storage compounds, they differ greatly in their endomembrane trafficking pathways. Starchy endosperm cells form prolamin protein bodies within the endoplasmic reticulum (ER), whereas aleurone cells accumulate lipid droplets in the ER and storage proteins, including prolamins, inside vacuoles (Larkins and Hurkman, 1978; Bowman et al., 1988; Reyes et al., 2011; Pedrazzini et al., 2016). While the starchy endosperm devotes a large portion of the ER to the stable accumulation of prolamin-rich protein bodies, aleurone cells transiently amass prolamin accretions in the ER which are then delivered to the vacuoles by an ATG8-independent autophagic mechanism (Reyes et al., 2011).

Autophagy plays important roles during development and stress protection by recycling nutrients needed for survival and new growth, and by removing damaged organelles, protein aggregates, and pathogens (Xiong et al., 2007; Li and Vierstra, 2012; Lv et al., 2014; Ren et al., 2014; Galluzzi et al., 2017). Two major autophagic routes have been identified: macro- and microautophagy. The best understood is macroautophagy, whereby a cup-shaped, double membrane structure called the phagophore expands and sequesters cytoplasmic constituents. It then seals to generate a double membrane-bound organelle called autophagosome which fuses with the vacuolar membrane/tonoplast to release its internal content as an autophagic body. Autophagy bodies and their cargo are then degraded by an assortment of vacuolar hydrolases, with the products then reused for metabolisms and new growth (Feng et al., 2014). During microautophagy, the tonoplast directly engulfs cytoplasmic material *via* invaginations. The resulting cargo-containing vesicles are then pinched off, released into the vacuole as autophagic bodies, and either stored or degraded (Sakai et al., 1998; Muller et al., 2000; Uttenweiler et al., 2005; Krick et al., 2008; Kawamura et al., 2012).

The molecular mechanisms underlying macroautophagy have been intensively studied in numerous eukaryotes, including plants, where over 50 proteins are likely involved (Xie and Klionsky, 2007; Li and Vierstra, 2012). Master regulators include several protein kinases responsive to the nutritional needs of the cell, mostly notably the TOR (target-of-rapamycin) kinase, which promotes the assembly of the downstream ATG1 (Autophagy Related 1) kinase complex (Ohsumi, 2001; Klionsky, 2007; Li et al., 2014; Pu et al., 2017). The ATG1 complex then initiates several events required for phagophore assembly, including: (i) addition of the signature lipid phosphatidylinostitol-3 phosphate to phagophore membrane by the phosphatidylinostitol-3 kinase complex VPS34, (ii) expansion of the phagophore mediated by ATG9, ATG2, and ATG18, and (iii) decoration of the membrane (both inner and outer leaflets) with ATG8 protein conjugated with the lipid phosphatidylethanolamine (PE). The ATG8-PE adduct then embeds within autophagic membranes, where it participates in phagophore expansion and maturation (Weidberg et al., 2011; Yu and Melia, 2017), selection of autophagic cargo through its interaction with cargo receptors (Zaffagnini and Martens, 2016), and fusion of autophagosomes with vacuoles (Nguyen et al., 2016). ATG8 is a member of the ubiquitin-fold protein family, and its ATP-dependent conjugation to PE requires the E1-like ligase ATG7, the E2-like ligase ATG3, and the E3-like ligase complex ATG12–ATG5-ATG16 (Romanov et al., 2012; Noda et al., 2013; Walczak and Martens, 2013; Kaufmann et al., 2014).

At present, much less is known about the molecular mechanisms underpinning microautophagy. In yeast and animals, either invaginations or arm-like protrusions of the vacuolar (or lysosomal) membrane participate in the uptake of cytosolic components, including mitochondria, peroxisomes, endosomes, ER, lipid droplets, and nuclear fragments (Sakai et al., 1998; Muller et al., 2000; Nowikovsky et al., 2007; Kawamura et al., 2012; Schuck et al., 2014; Tsuji et al., 2017). Only a few cases of selective microautophagy have been reported for plants, such as the vacuolar sequestration of cytoplasmic anthocyanin aggregates in *Arabidopsis thaliana* epidermal cells (Chanoca et al., 2015) and the selective removal of chloroplasts damaged by high light (Nakamura et al., 2018), but at present no microautophagy-specific factors have been identified. Thus far, microautophagy in yeast, animals, and plants has been reported to require (Muller et al., 2000; Uttenweiler et al., 2007; Farre et al., 2008; Krick et al., 2008; Nakamura et al., 2018) or be independent of Chanoca et al. (2015) and Oku et al. (2017) the core ATG machinery, including ATG8.

Our prior studies showed that the delivery of prolamin storage proteins to the vacuole is an unconventional autophagy route in plants because it deposits prolamin-filled ER domains into vacuoles without needing either the formation of typical autophagosomes or ATG8 and its lipidation pathway (Reyes et al., 2011; Li et al., 2015). However, the underpinning cellular and molecular processes that mediate this transport are currently unknown. Here, we studied the vacuolar trafficking of prolamins in maize aleurone cells by electron tomography and immunolabeling and found it to be mediated by microautophagy (*i.e.*, direct engulfment of cytoplasmic contents by vacuoles), which appears to selectively sequester ER domains containing storage protein bodies. By comparing gene expression in aleurone vs. starchy endosperm and identifying proteins associated with the aleurone tonoplast, we identified 10 candidate proteins that could help mediate microautophagy of prolamins, including a phospholipase D and a lectin-like protein. We found that both could remodel the tonoplast when transiently expressed in *Arabidopsis* leaf protoplasts and preferentially localized to the tonoplast in maize aleurone cells at the sites of prolamin protein body uptake, thus supporting their roles in the microautophagic sequestration of storage proteins in the maize aleurone.

## Materials and Methods

### Plant Materials and Growth

Maize plants (*Zea mays*, inbred B73) used for RNA-sequencing were grown in a greenhouse supplemented with 700 μmol m^–2^ s^–1^ white light under a 14-h-light/10-h-dark photoperiod, with the average temperature set at 28°C during the day and 21°C at night. Maize B73 plants used for qRT-PCR analysis were grown at West Madison Agricultural Research Station (University of Wisconsin-Madison) during June-August 2016. Maize plants (inbred W22) used for electron microscopy were grown in greenhouses of Wisconsin Crop Innovation Center (University of Wisconsin-Madison), Walnut Street Greenhouse (University of Wisconsin-Madison), and at West Madison Agricultural Research Station at different times of 2016 and 2017. Maize plants (inbred W22) used for co-immunoprecipitation and proteomic analysis were grown at West Madison Agricultural Research Station during June-August 2018. Maize plants (Hi-II (Armstrong, 1991)) producing kernels for *in vitro* culture and bombardment of endosperm were grown in the greenhouse of the Wisconsin Crop Innovation Center. Maize grown at Wisconsin Crop Innovation Center was under a 16-h-light/8-h-dark photoperiod, with supplemental lighting provided at an intensity of 330 μmol m^–2^ s^–1^ at ∼1.7 m below the lights, and average temperatures of 28°C during the day and 21°C at night. Maize grown at Walnut Street Greenhouse supplemented with 500 μmol m^–2^ s^–1^ white light under a 16-h-light/8-h-dark photoperiod, at temperature of 26.7 ± 2.7°C.

### Electron Microscopy and Immunogold Labeling

Slices of endosperm tissue from developing kernels at different developmental stages were high-pressure frozen in an ICE high pressure freezer (Leica) and freeze-substituted in 2% OsO_4_ in anhydrous acetone at –80°C overnight followed by slow warming to room temperature over a period of 6 h. Samples were rinsed in acetone and infiltrated in Epon resin (Ted Pella). Sections were stained with 2% uranyl acetate in 70% methanol followed by Reynold’s lead citrate (2.6% lead nitrate and 3.5% sodium citrate, pH 12.0), and observed in a FEI CM120 electron microscope.

For immunogold labeling, high-pressure frozen endosperm samples were substituted in 0.2% uranyl acetate (Electron Microscopy Sciences) and 0.2% glutaraldehyde (Electron Microscopy Sciences) in acetone at –80°C for 72 h and warmed to –50°C for 24 h. Samples were infiltrated with Lowicryl HM20 (Electron Microscopy Sciences) and polymerized at –50°C under UV light. Sections were mounted on formvar-coated nickel grids and blocked for 20 min with a 10% (w/v) solution of nonfat milk in Tris-buffered saline (TBS) (pH 7.4) containing 0.1% Tween 20. The sections were incubated with primary rabbit antibodies against 15-kD β-zein, legumin-1 (Woo et al., 2001) or *Arabidopsis* AVP1 (Paez-Valencia et al., 2011) diluted 1:10 in TBS-Tween 20 for 1.5 h, rinsed in TBS containing 0.5% Tween 20, and transferred to the secondary antibody (anti-rabbit IgG 1:10) conjugated to 15-nm gold particles for 1 h. Controls were performed by using pre-immune rabbit sera.

### Electron Tomography

Semi-thick sections (250 nm) from Epon-embedded endosperm samples were imaged in a FEI Tecnai TF30 300-kV intermediate-voltage electron microscope between 60° and –60° angles, at 1° angle intervals about two orthogonal axes (Mastronarde, 1997). Images were collected using a US1000 Gatan camera at a pixel size of 0.715 nm. Tomograms were calculated using simultaneous iterative reconstruction technique (SIRT) (Gilbert, 1972), and the two single-axis tomograms was merged as previously described (Mastronarde, 1997). Tomograms were displayed and analyzed with 3Dmod, the graphic component of the IMOD software package (Kremer et al., 1996). The thinning factor for each tomogram was calculated and corrected for in the models.

### RNA Extraction and RNA-Seq Library Construction and Sequencing

Maize kernels from two plants (inbred B73) were collected at 18 DAP and 22 DAP, respectively. The kernels were frozen to −80°C, defrosted, and then the aleurone cell layer and starchy endosperm were immediately excised by mechanical peeling. Each tissue set was carefully rinsed using distilled water before further processing. For constructing and sequencing RNA-seq libraries, RNA was extracted from the collected aleurone and starchy endosperm samples. Tissues were frozen in liquid nitrogen and ground in NTES buffer (20 mM Tris-HCl, pH 8.0, 100 mM NaCl, 10 mM EDTA, and 1% SDS) to avoid starch solubilization. Nucleic acids were extracted three times using Tris-buffered phenol/chloroform (1:1), pH 8, followed by clarification at 10,000 × *g*. The RNA present in the aqueous phase was later purified using Trizol reagent (Invitrogen) according to the manufacturer’s instructions. One microgram of total RNA was reversely transcribed to the first strand of the cDNA using oligo(dT) in a 20-mL reaction volume using AMV transcriptase (Promega). RNA quality was checked using Agilent Bioanalyzer for one of each pair of biological replicates and all RNA samples had RNA integrity number ≥7.6, which is close to Illumina’s recommendation of RNA integrity number ≥8. RNA-Seq libraries were made according to the TruSeq RNA protocol; 1 μg of total RNA was used for all of libraries (mean size approximately 300 bp +/− 30 bp). The samples were sequenced using indexed adapters that allowed to pool four libraries in a single lane of an Illumina GA sequencer to produce 51-nucleotide single-ended reads.

### Read Mapping and Calculation of Transcript Abundance

The RNA-seq reads for aleurone and starchy endosperm at 18 and 22 DAP were quality checked using fastqc software^1^. Low quality bases (quality score < 20) were trimmed from the reads, and only reads with lengths ≥30 bases were aligned to maize B73 version 3 genome (B73 RefGen_v3) (Hubbard et al., 2002) and version 3.26 gene models of 39,465 high confidence protein coding genes using RSEM (RNA-seq by Expectation Maximization) (Li and Dewey, 2011). During the alignment of all the RNA-seq datasets analyzed in this paper, the RSEM program first used Bowtie2 version 2.2.3 (Langmead and Salzberg, 2012) to align the RNA-sequencing reads to exons and SAMtools version 0.1.19-44428cd (Li et al., 2009) to sort the aligned reads, and then estimated the mRNA abundance based on transcripts per million (TPM) (Li and Dewey, 2011). For RNA-seq data of aleurone and starchy endosperm samples, sequencing fragment-length mean and standard deviation were provided to RSEM.

RNA-seq data previously generated for aleurone and starchy endosperm at 8 DAP (Zhan et al., 2015) and 15 DAP (Yi et al., 2015) were downloaded from National Center for Biotechnology Information (NCBI) SRA database. All downloaded RNA-seq dataset consisted of three biological replicates, which were also quality checked and trimmed as done for aleurone and starchy endosperm (18 and 22 DAP) datasets. We used the RNA-seq dataset of 8 DAP from the central starchy endosperm (CSE) to represent the transcriptome of starchy endosperm at that time point. All downloaded RNA-sequencing data were aligned to the same reference genome using RSEM as previously described for aleurone and starchy endosperm (18 and 22 DAP) RNA-sequencing data. The RNA-sequencing of aleurone and starchy endosperm at 8 and 15 DAP was performed using pair-ended reads; unfortunately, because RSEM could not accept discordant alignment, all datasets from 8 DAP tissues had very low mapping rates. To generate mapping rates comparable to those in the original study, all pair-ended reads of 8 DAP datasets were aligned as single-ended reads, *i.e*., only mate one in each paired reads were used for alignment. The fragment-length mean and standard deviation were set to 300 and 100 bp based on information obtained by personal communication with the authors. Gene expressions were reported as TPM, using the average value of all available biological replicates as the TPM of a gene if, and only if, all biological replicates have TPM > 0. Otherwise, the gene was treated as not expressed (TPM = 0).

### Gene Differential Expression Analysis

After sequence alignment, we obtained the read counts for all protein coding genes using HTSeq (Anders et al., 2014) and identified differentially expressed genes (DEGs) in aleurone vs. starchy endosperm at 18 and 22 DAP using paired tests of edgeR (Robinson et al., 2010) and limma (Ritchie et al., 2015). After calculating the false discovery rates (FDR) from *p*-values, the significance cutoff of DEGs was set to FDR ≤ 0.05. To identify DEGs with high confidence, we used only those calculated by both edgeR and limma (Supplementary Table 2) with the detailed settings for each described below.

Read counts of each gene were obtained using HTSeq 0.6.1 (Anders et al., 2014) and the feature used for counting was “gene” in the gene annotation file (GFF file). Then, paired-tests were conducted using R package edgeR 3.8.2 (Robinson et al., 2010) and limma 3.32.10 (Ritchie et al., 2015) to compare the gene expression levels of aleurone vs. starchy endosperm at 18 DAP and 22 DAP, respectively, where corresponding biological replicates of aleurone and starchy endosperm were paired. EdgeR and limma use different methods to model mean-variance relationship of read counts of all genes. EdgeR models read counts with a negative binomial distribution assuming a quadratic mean-variance relationship. Limma first transforms raw read counts into log2-counts per million (log-CPM) and models the mean-variance relationship of log-CPM of all genes by linear modeling and calculating precision weights, assuming that log-CPM is normally distributed. We applied both programs to identify DEGs with high confidence, *i.e.*, DEGs identified by both edgeR and limma calculations. Multiple test correction of *p*-values was done by calculating a FDR for each *p*-value using the Bioconductor’s qvalue package in R^2^. Additionally, we eliminated up-regulated and down-regulated (aleurone vs. starchy endosperm) for which the estimated TPM = 0 in one sample due to low abundance of their transcripts captured by RNA-seq.

The process of gene differential analysis with edgeR and limma is briefly described here. Normalization of the sequencing depth was conducted in edgeR with calcNormFactors. The design and contrast matrices were set up to perform differential expression tests on paired biological replicates of aleurone and starchy endosperm. For all paired-tests, the mean-variance relationship of RNA-seq reads was modeled with generalized linear models (GMLs) to reduce the number of false positive DEGs caused by underestimation of gene expression variance. Specifically, estimateGLMCommonDisp, estimateGLMTrendedDisp, and estimateGLMTagwiseDisp were applied to read counts of all RNA-seq samples sequentially after normalization factors had been calculated. Lastly, DEGs were determined using a GLM likelihood ratio test with functions glmFit and glmLRT (McCarthy et al., 2012). Normalization of the sequencing depth was conducted with edgeR’s calcNormFactors function. Then, the design and contrast matrices were set up to perform differential expression tests on paired biological replicates of aleurone and starchy endosperm same as with edgeR. Then mean-variance relationship of RNA-seq data was modeled by the voom function of limma to remove heteroscedascity from count data (*i.e.*, the variability of variance is unequal across the range of mean). To compare gene expression levels, linear modeling was performed by the lmFit and contrasts.fit functions, and empirical Bayes moderation was performed by the function eBayes which estimates DEGs of interest by taking account of gene-wise variability across all genes (Smyth, 2004).

### Reverse Transcription Quantitative Polymerase Chain Reaction Validation of Differentially Expressed Genes

RNA was isolated from maize kernels (inbred B73) harvested at 18 DAP and 22 DAP, respectively, as described before. Reverse transcription was performed using the High-Capacity cDNA Reverse Transcription Kit with RNase inhibitor (Thermo Fisher Scientific). Amplification and detection were conducted on a Stratagene 512 MX3000P qPCR system to monitor double strand (ds) DNA synthesis. All reactions contained 2 μl of cDNA (∼250 ng/μl), 3 μL of each of the two gene-specific primers (2 μM), and 10 μl of MAXIMA SYBR Green/ROX qPCR Master Mix (Thermo Fisher Scientific) in a final volume of 20 μl, and were performed with a 60°C annealing temperature. Results were analyzed with LinRegPCR (version 2013.0^3^). The relative value for expression levels of each gene was calculated by the comparative Ct method (Schmittgen and Livak, 2008) using the *Ubc9* gene as reference. The fold changes (aleurone vs. starchy endosperm) of each DEG were obtained by calculating the ratio of the relative expression in aleurone vs. in starchy endosperm from the mean of three biological replicates, each analyzed in triplicate. Primers used for quantitative PCR analysis have amplification efficiencies between 90 and 110% and are listed in Supplementary Table 9.

### Aleurone Tonoplast Isolation, Co-immunoprecipitation, and Immunoblot Analysis

Maize kernels were collected at 20 DAP, frozen in liquid nitrogen, and stored at −80°C. For tonoplast isolation. Approximately 100 mg of aleurone tissue was excised from three biological replicates from thawed kernels by mechanical peeling and pulverized in the 1:2 (w/v) homogenization buffer (250 mM sorbitol, 50 mM HEPES-BTP [pH 7.4], 6 mM EGTA, 1.2% [w/v] polyvinil porrolidone-40 [Sigma-Aldrich], 1× protease inhibitor cocktail complete [Roche]). All steps were performed on ice or at 4°C unless specified otherwise and in low protein-binding tubes. The resulting crude extracts were filtered through two layers of Miracloth and clarified at 10,000 × *g* for 15 min to remove starch grains, unbroken cells, cell wall fragments, and nuclei. The supernatant was centrifuged at 60,000 × *g* for 30 min, and the resulting pellet (microsomal fraction) was suspended in 200 ml of the solubilizing buffer (25 mM HEPES-BPT [pH 7.4], 150 mM NaCl, 1 mM EDTA, 10% Glycerol, 1% NaF, 1% NP40, 1 mM PMSF, 1× protease inhibitor cocktail Complete [Roche]). This fraction was layered over a dextran step gradient consisting of 1% (w/w) (top) and 8% (w/w) (bottom) dextran T-70. Tonoplast membranes were collected at the 1 to 8% dextran interface. The membranes were standardized by protein concentration (250 mg in μl aliquots) as determined using the bicinchoninic acid (BCA) assay and bovine serum albumin as standard.

Immunoprecipitation with anti-AVP1 antibodies (Paez-Valencia et al., 2011) was performed from both the microsomal and tonoplast fractions. To help eliminate proteins binding non-specifically to the beads, each sample was incubated for 1 h with 25 μl of protein A/G magnetic beads alone (NEB BioLabs), and the beads then removed by centrifugation. Twenty μl of either anti-AVP1 polyclonal antibodies or pre-immune serum were then added to the samples, and incubated for 1 h under gentle agitation, followed by a 1 h incubation with 25 μl of magnetic beads. Beads were collected, washed three times with fresh pre-chilled solubilizing buffer, resuspended in 30 μl of sample buffer (25 mM HEPES-BPT [pH 7.4], 8 M urea, 100 mM NaCl, 10% glycerol), and incubated at 70°C for 5 min. Proteins from the supernatant were precipitated using methanol and chloroform (Wessel and Flugge, 1984) for shotgun mass spectrometry. To confirm the enrichment of H+-PPases, the crude extract, and immunoprecipitates of microsomal fraction and tonoplast fractions were subjected to SDS-PAGE analysis followed by immunoblotting with anti-H+-PPase antibodies (Paez-Valencia et al., 2011).

### Tandem Mass Spectrometry and Protein Quantification of Proteins Co-immunoprecipitated With Anti-H+-PPase Antibodies

Co-immunoprecipitated proteins from the tonoplast fraction were eluted using 100 μl of 8 M urea, reduced in 10 mM dithiothreitol (DTT) at room temperature for 1 h, and then alkylated in 50 mM 2-iodoacetamide in the dark at room temperature for 1 h. After quenching excess alkylating agent with 50 mM DTT for 5 min, samples were diluted with 900 μl of 25 mM (NH_4_)HCO_3_ and then digested with 0.5 μg sequencing grade trypsin (Promega) at 37°C for 18 h. The resulting peptides were then vacuum dried to a final volume of approximately 250 μL, acidified with 10% trifluoroacetic acid (TFA) to reduce the pH to below 3.0, and then desalted and concentrated on a 100 μl Bond Elut OMIX C18 pipette tip (Agilent Technologies) according to the manufacturer’s instructions. The samples were eluted in 50 μl of 75% acetonitrile and 0.1% acetic acid, lyophilized, and then resuspended in 50 μl of 5% acetonitrile and 0.1% formic acid. Nano-scale liquid chromatography (LC) separation of tryptic peptides was done on a Dionex Ultimate™ 3000 Rapid Separation LC system (Thermo Fisher Scientific). The protein digests were loaded onto a 20 μL nanoViper™ sample loop (Thermo Fisher Scientific) and separated on a C18 analytical column (Acclaim PepMap™ RSLC C18 column, 2 μm particle size, 100 Å pore size, 75 μm × 25 cm [Thermo Fisher Scientific]) by applying a linear 2 h gradient from 4 to 36% acetonitrile in 0.1% formic acid, with a column flow rate of 250 nl/min. The eluded tryptic peptides were analyzed with a Q Exactive Plus mass spectrometer (Thermo Fisher Scientific) possessing a Nanospray Flex Ion source (Thermo Fisher Scientific) fitted with a stainless-steel nano-bore emitter operated in positive electro-spray ionization mode at a capillary voltage of 1.9 kV. Data-dependent acquisition of full MS scans within a mass range of 380–1,500 m/z at a resolution of 70,000 was performed with the automatic gain control (AGC) target set to 3 × 10^6^ and the maximum fill time set to 200 ms. High energy collision-induced dissociation fragmentation of the top eight most intense peaks was done with a normalized collision energy of 28, an intensity threshold of 1.3 × 10^4^ counts, and an isolation window of 3.0 m/z, excluding precursors that had an unassigned, +1, +7, or +8 charge state. MS/MS scans were conducted at a resolution of 17,500 with an AGC target of 2 × 10^5^ and a maximum fill time of 300 ms. Dynamic exclusion was performed with a repeat count of two and an exclusion duration of 30 s. The minimum MS ion count for triggering MS/MS was set to 4 × 10^3^ counts.

The MS/MS spectra were analyzed using Proteome Discoverer software (version 2.0.0.802, Thermo Fisher Scientific), searching the *Zea mays* B73 proteome database (Zea-mays.AGPv3.21.pep.all from^4^). Peptides were assigned using SEQUEST HT (Eng et al., 1994), with search parameters set to zero and one missed cleavage from the trypsin digestion, a minimum peptide length of six, a precursor mass tolerance of 10 ppm, and a fragment mass tolerance of 0.02 Da. Carbamidomethylation of cysteines was specified as a static modification and oxidation of methionines and N-terminal acetylation were specified as dynamic modifications. A target FDR of 0.01 (strict) was used as validation for peptide-spectral matches (PSMs) and peptides. Proteins containing similar peptides which could not be differentiated based on the MS/MS analysis alone were grouped to satisfy the principles of parsimony. Label-free quantification was performed based on the universal signal response factor (Silva et al., 2006) using a minimum Quan value threshold of 0.0001 for unique peptides and “3 Top N” peptides for area calculation, with the relative abundances for the full-length proteins being generated from the averages of 3 biological replicates.

### Gene Annotation, BLAST, and Enrichment Analyses

Maize gene names and functional description of the protein products were obtained from EnsemblPlants^5^. To infer functional descriptions of proteins without any functional annotation, we compared by NCBI-BLAST-2.2.30+ (Camacho et al., 2009) the peptide sequences of the maize protein model set 5b+ for RefGen_v3 (Hubbard et al., 2002) against the *Arabidopsis thaliana* representative protein models (TAIR10). The expectation value (*E*-value) threshold was set to 1E-5 and the best hit in *Arabidopsis* was considered for each maize protein as its putative homolog.

Gene ontology (GO) enrichment analyses were conducted with the Maize-GAMER GO ontology annotations for v3 genome provided on AgriGOv2 (Tian et al., 2017) and the topGO package in R, using only protein-coding loci. The enrichments were performed with the “weight01” algorithm and Fisher’s exact tests for the subcellular localizations (*i.e.*, the cellular component aspect of GO) of the proteins of interests. FDRs were calculated from the *p*-values using p.adjust function in R with the “BH” algorithm (Benjamini and Yekutieli, 2001). For GO enrichment analyses of proteins detected in the co-immunoprecipitations, we used version 3.26 gene models which encodes 39,465 representative proteins (one protein for each gene) (Supplementary Table 2). For proteins detected in the co-immunoprecipitates with anti-H+-PPase and pre-immune sera, we included all the proteins detected in at least one biological replicate. Using the topGO package, we obtained the list of enriched GO terms and their FDRs for proteins detected in the co- immunoprecipitates (Supplementary Tables 6,7). To eliminate proteins potentially captured by non-specific interactions, we first added one to all protein abundance values to avoid denominators of 0 and calculated enrichment fold differences detected in co-immunoprecipitations with anti- H+-PPase antibodies vs. pre-immune serum. The higher the enrichment ratio, the more likely the protein was captured by specific interaction with anti- H+-PPase antibodies. The thresholds of enriched proteins (high- and medium- confidence) were defined as follow: high confidence: at least two out of three biological replicates have fold differences ≥ 2; medium confidence: only one biological replicate has fold differences ≥ 2, and the protein is never detected in pre-immune serum; low confidence: the remaining identified proteins.

To calculate the *p*-value and fold enrichment of putative tonoplast proteins in maize based on sequence homology to a published *Arabidopsis* tonoplast proteome, we used the Fisher’s exact test (R’s fisher.test() function with “alternative” set to “great”).

### cDNA Cloning, Constructs, and Plasmids

For transforming *Arabidopsis* mesophyll protoplasts and developing maize endosperm, all cDNAs sequences were cloned into the pRTL2-mCherry vector (*Arabidopsis* Biological Resource Center, stock number CD3-1062). The cDNAs of all the microautophagy candidates were fused directly to the 5′ end of the coding region for mCherry.

### Transformation and Imaging of *Arabidopsis* Protoplasts and Co-localization Analysis Microautophagy Candidates

*Arabidopsis* protoplasts from leaves of transgenic plants expressing *pUBQ10::VAMP711-YFP* (Geldner et al., 2009) was isolated as previously described (Wu et al., 2009). Briefly, abaxial epidermis were removed from young leaves of 2–3 week old plants by the “Tape-*Arabidopsis* Sandwich” technique (Wu et al., 2009) to expose the mesophyll cells. After cultivating the leaves in the enzyme buffer for ∼1 h, the protoplasts were harvested by centrifuging at 100 × *g*, washed twice with pre-chilled modified W5 solution, rested on ice for 30 min, and resuspended in the MMG solution (4 mM MES, 0.4 M mannitol and 15 mM MgCl_2_ at pH 5.7) before transformation (Wu et al., 2009). Approximately 12 μg of pRTL2 vectors were used to transform 100 μL of protoplasts in MMG solution. The vectors were dissolved in freshly prepared 40% (w/v) PEG (Wu et al., 2009) and gently mixed on ice for 10–15 min with protoplasts and then incubated at 23°C in darkness for 14 h before confocal microscopic analysis. Protoplast transformation was repeated three times.

The protoplasts were loaded onto an 18 Well Flat μ-Slide (Ibidi), and imaged with a 780 Zeiss laser scanning confocal microscope using a 63× either water or oil immersion objective (N.A. 1.46). To reduce noise, the multitrack mode was used for sequentially imaging of YFP, mCherry, and chlorophyll. YFP was excited with a 514 nm laser line and detected with a 519–562 nm band-pass filter, mCherry was excited with a 561 nm laser line and detected with a 579–633 nm band-pass filter, and chlorophyll was excited with a 633 nm laser line and detected with a 660–721 nm band-pass filter. The emission spectra of YFP and mCherry were confirmed by spectral scans. To characterize the membrane morphology of tonoplast, confocal images were captured at different depth, including cortical and middle plane views. Coloc2 in FijiFor were used fpr co-localization analyses of VAMP711-YFP with mCherry-tagged microautophagy candidates (Schindelin et al., 2012). We selected the vacuole as region of interest (ROI) by tracing outside the contour of tonoplast labeled by VAMP711-YFP.

### *In vitro* Culture, Biolistic Bombardment, and Imaging of Developing Maize Endosperm

Excised maize endosperms were cultured and transformed according to published protocols (Ding et al., 2021). Briefly, we excised developing endosperms from Hi-II ears at 8 DAP and placed them on solid medium containing 4.3 g/l Murashige and Skoog basal salt, 0.5% v/v Murashige Skoog vitamins stock solution, 5 mg/l thiamine HCl, 400 mg/l Asn, 10 μg/L 6-benzylaminopurine, 15% sucrose (pH 5.8), g/l of gelrite, and 500 μg/ml carbenicillin. Plates were wrapped with aluminum foil and kept at 28°C for 2 days. Gene delivery involved the PDS-1000/He Biolistic Particle Delivery System in combination with 1.0 μm gold microparticles (Bio-Radz). For each bombardment, we used approximately 500 ng of gold nanoparticles coated with a total of 1.5 μg of pRTL2 and/or 1–1.5 μg of the binary vector containing the TIP1-YFP expression cassette (Krishnakumar et al., 2015). The DNA-coated gold nanoparticles were fired under 23–25 in Hg (11.3–12.3 psi) vacuum and with 1,100-psi rupture disk (Bio-rad). Each endosperm plate was bombarded once, wrapped with aluminum foil, and put back into the growth chamber.

Confocal imaging of transfected aleurone cells was performed at approximately 24, 48, 72, and 96 h after bombardment. To label the vacuolar lumen, we vacuum-infiltrated the endosperms for 3 min with 10 μM BCECF-AM (Invitrogen), rinsed them, and placed them back on plates for 30 min to let BCECF-AM reach the vacuoles (Swanson and Jones, 1996). We prepared thin, paradermal sections of endosperms in MilliQ-water and place them between two cover glasses for confocal imaging. Aleurone cells transiently expressing fluorescently tagged proteins were visualized with a 780 Zeiss laser scanning confocal microscope using a 63x water or oil immersion objective (N.A. 1.46). YFP and mCherry was detected as explained above for protoplasts. BCECF was excited with a 488 nm laser line and detected with a 517–588 nm band-pass filter, and storage proteins were excited with 405 nm wavelengths and detected with a 459–500 nm band-pass filter. The fluorescence signals of YFP, mCherry, and BCECF were confirmed by spectral scans. To characterize the shape and size of vacuoles and tonoplast morphology, we used z-stack images that were captured different depths of aleurone cells.

## Results

### Endoplasmic Reticulum Storage Protein Bodies Are Delivered to Aleurone Vacuoles by Microautophagy

Our previous studies on storage protein trafficking in the maize aleurone revealed that prolamins (zeins) are delivered to protein storage vacuoles through an autophagy-type route that does not involve either ATG8 lipidation or assembly of autophagosomes (Reyes et al., 2011; Li et al., 2015). Here, we studied this process in more detail by conventional and 3D electron microscopy of high-pressure frozen/freeze-substituted developing endosperm tissues harvested between 15 and 20 days after pollination (DAP), a developmental window when maize aleurone cells actively synthesize storage proteins (Reyes et al., 2011).

**FIGURE 1:**
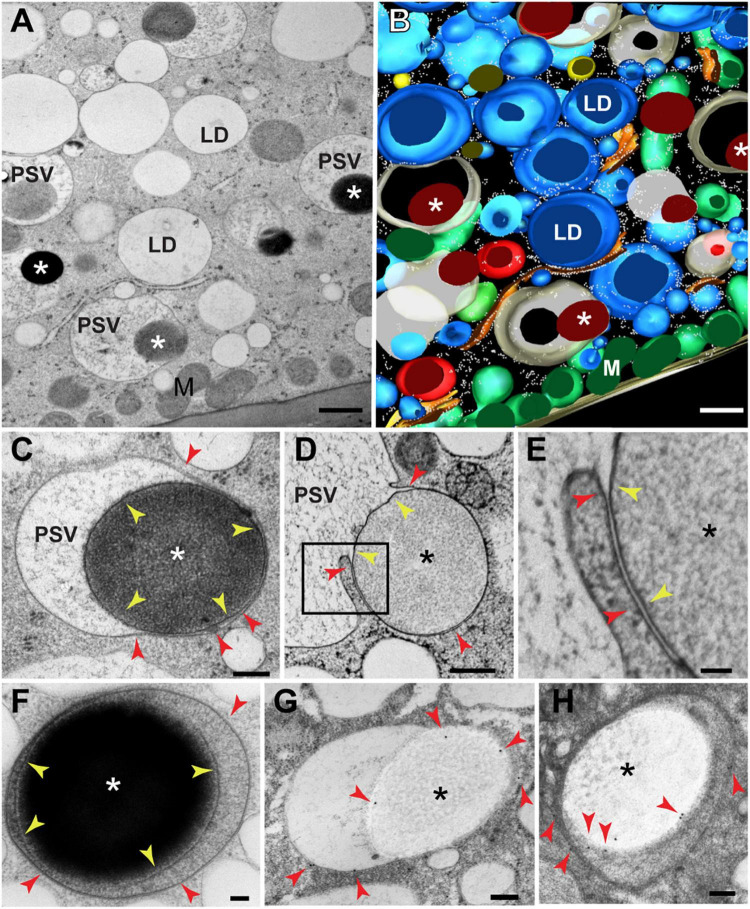
Transmission electron microscopy of protein storage vacuoles (PSVs) in maize aleurone cells. **(A)** Individual serial section and **(B)** 3D reconstruction resulting from 8 serial sections (70 nm thick) of wild type (W22) maize aleurone cells at 20 DAP. Note the presence of large electron-dense inclusions (asterisks) inside vacuoles. **(C)** Detail of a developing vacuoles with a protein storage inclusion (asterisk) surrounded by a membrane (yellow arrowheads). The vacuolar membrane or tonoplast is indicated by red arrowheads. **(D,E)** Another example of a vacuole at 24 DAP engulfing a protein storage inclusion (asterisk). The membrane domain surrounding the inclusion is indicated by yellow arrowheads and the membrane domain continuous with the rest of the tonoplast, with red arrowheads. **(F)** Aleurone vacuoles at 26 DAP containing a storage protein inclusion (I) surrounded by a membrane (yellow arrowheads). **(G,H)** Immunolabeling with antibodies against a tonoplast-resident H^+^-pyrophosphatase revealed that the membrane domain surrounding the inclusion derives from the tonoplast. Red arrowheads indicate gold particles. LD, lipid droplet; M, mitochondrion. Scale bar = 800 nm in **(A,B)**; 500 nm in **(D)**; 200 nm in **(C,G,H)**; 100 nm in **(E,F)**.

At 20 DAP, aleurone vacuoles were fairy uniform in size (vacuolar diameter = 1.5 μm ± 0.2 μm; *n* = 15 vacuoles) and contain a few large storage protein inclusions 1–1.5 mm in diameter and displaying variable electron density (indicated by asterisks in Figures 1A,B and Supplementary Figures 1A,B). Closer examination showed that these inclusions are enclosed by membranes (Figures 1C–F, yellow arrowheads), with the majority almost exclusively found within vacuoles and in close association with the tonoplast (Figures 1C–F, red arrowheads). In some cases, the tonoplast was partially appressed to the membrane surrounding the protein inclusion as expected for a recent engulfment event (Figures 1C,D). To determine the origin of the membranes surrounding these inclusions, we performed immunogold labeling with antibodies against a tonoplast-resident pyrophosphatase (Paez-Valencia et al., 2011), and confirmed that they were indeed of tonoplast origin (Figures 1G,H and Supplementary Figure 1C). Taken together, we concluded that protein bodies that form in the ER of aleurone cells (Reyes et al., 2011) are directly engulfed *via* microautophagy.

**FIGURE 2:**
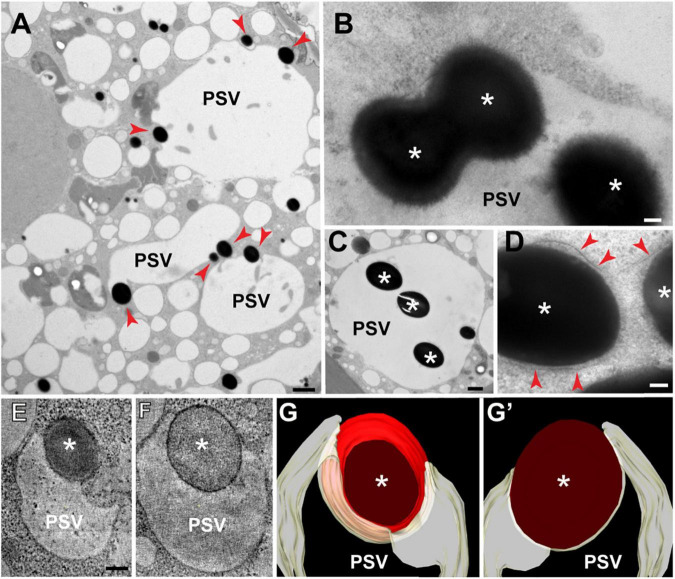
Transmission electron microscopy of storage protein sequestration by microautophagy in maize aleurone cells. **(A–D)** Aleurone cells at 15 DAP with developing protein storage vacuoles (PSVs). **(A)** Storage protein inclusions (asterisks) indicated by red arrowheads at the periphery of PSVs. **(B)** Two inclusions (asterisks) merging inside a vacuole. **(C)** Vacuole with multiple inclusions. **(D)** Storage protein bodies (asterisks) surrounded by ER membrane (arrowheads) still in the cytoplasm. **(E–G′)** Electron tomographic analysis of a vacuole engulfing a storage protein body (asterisk) at 20 DAP. **(E,F)** Two tomographic slices at different depths of the same vacuole. **(G,G′)** Two views of the tomographic reconstruction of the PSV and inclusion from the same tomogram. Scale bars = 1 μm in **(A,C)**; 100 nm in **(B,D–G′)**.

To visualize earlier stages of the vacuolar sequestration of storage protein bodies, we examined aleurone cells at 15 DAP when vacuoles are larger and more variable in size (vacuolar diameter = 4.7 μm ± 2 μm; *n* = 15 vacuoles). Numerous storage protein bodies were partially or fully incorporated within single vacuoles (Figures 2A–C), with some inclusions appearing to merge into larger versions within the vacuolar lumen (Figure 2B). We also detected protein bodies in the ER as evidenced by the presence of surface ribosomes (red arrows in Figure 2D). To better visualize the 3D organization of the tonoplast at sites of protein body engulfment, we performed electron tomography of aleurone cells at 20 DAP. Here, intermediate structures were seen where the tonoplast partially surrounds a membrane-limited protein body (Figures 2E–G′), consistent with the sequestration of storage protein bodies into aleurone vacuoles by microautophagy.

**FIGURE 3:**
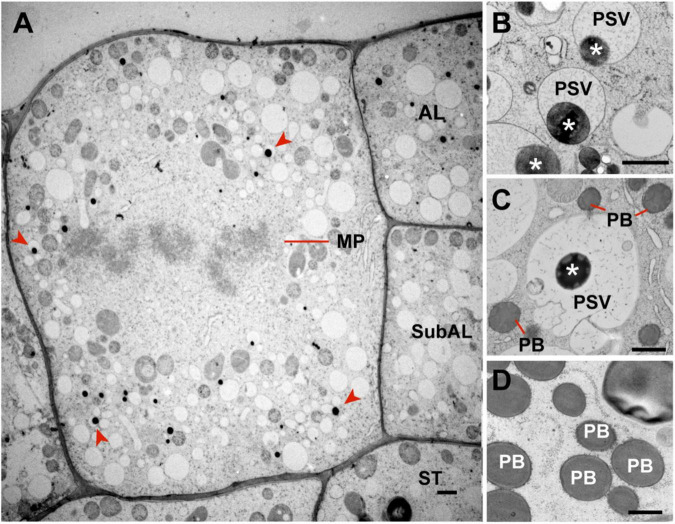
Occurrence of microautophagy in endosperm cells at 17 DAP. **(A)** Aleurone cell undergoing mitosis. Periclinal cell divisions in the aleurone layer give rise to a new aleurone (AL) and a sub-aleurone (Sub-AL) cell. Note that this dividing aleurone cell contains vacuoles with storage protein inclusions (red arrowheads). **(B)** Protein storage vacuoles (PSVs) with storage protein inclusions (asterisks) in aleurone cells. **(C)** Sub-aleurone cells containing both PSVs with protein inclusions (asterisks) and ER protein bodies (PBs). **(D)** ER protein bodies (PB) in starchy endosperm cells. MP, metaphase plane. Scale bars = 1 μm.

Microautophagy has not been fully characterized in plants, but our studies on the trafficking of storage proteins in maize aleurone cells (Reyes et al., 2011) and anthocyanin aggregates in *Arabidopsis* (Chanoca et al., 2015) implied a route independent of the ATG8 lipidation, in contrast to the microautophagy of photodamaged chloroplasts that depends on ATG8 lipidation (Nakamura et al., 2018). Consequently, the maize endosperm offers a unique opportunity to uncover novel microautophagy components given that the ER produces storage protein bodies in both aleurone and starchy endosperm cells, but only aleurone cells deliver them to vacuoles by microautophagy. Typically, differentiating aleurone cells divide periclinally (parallel to the organ surface) to generate a new cell (AL in Figure 3A) at the endosperm surface and a sub-aleurone cell (subAL in Figure 3A). Newly formed sub-aleurone cells first display both starchy endosperm and aleurone characteristics, but eventually differentiates into a starchy endosperm cell (ST in Figure 3A; Becraft and Asuncion-Crabb, 2000; Gruis et al., 2006). We observed cases of aleurone periclinal divisions (Figure 3A, evidenced by the metaphase plane, MP), aleurone cells with storage protein inclusions in developing vacuoles (Figure 3B), and sub-aleurone cells transitioning into starchy endosperm cells. During this transition, sub-aleurone cells contained both vacuoles with storage protein inclusions (likely formed when the cell was part of the aleurone layer) and typical ER protein bodies (Figure 3C). By contrast, fully differentiated starchy endosperm cells did not contain vacuoles with protein inclusions (Figure 3D), suggesting that the reprograming of aleurone cells to a starchy endosperm fate stabilizes ER protein bodies and/or removes the ability to undergo microautophagy.

### Transcriptomes of Aleurone and Starchy Endosperm Cells at 18 Days After Pollination and 22 Days After Pollination

The factors responsible for ATG8 lipidation-independent microautophagy remain to be identified in plants (Chanoca et al., 2015). Consequently, we reasoned that such factors might emerge upon comparing the aleurone and starchy endosperm transcriptomes, given the distinct mechanism used by aleurone and subaleurone cells to traffic ER-derived protein bodies. To identify genes specifically expressed at these important developmental stages, we manually dissected aleurone and starchy endosperm regions at 18 DAP and 22 DAP and performed deep transcriptome analysis by RNA-sequencing (RNA-seq). Overall, there were more genes expressed [transcript per million (TPM) > 0] in aleurone than in starchy endosperm cells (Supplementary Tables 1,2), with 19,359 and 18,907 genes having TPM > 0 in the aleurone samples at 18 and 22 DAP, respectively, and 13,022 and 8,661 genes having TPM > 0 in the starchy endosperm samples at 18 and 22 DAP, respectively. The differences were not caused by variations in sequencing depth as the number and percentage of sequence-aligned reads were similar between the two tissue samples (Supplementary Table 1). In addition, the correlations between biological replicates measured by Spearman correlation coefficients of log(TPM+1) were between 0.7 to 0.9 (Supplementary Table 1), indicating good reproducibility of RNA-sequencing and sequence alignment.

**FIGURE 4:**
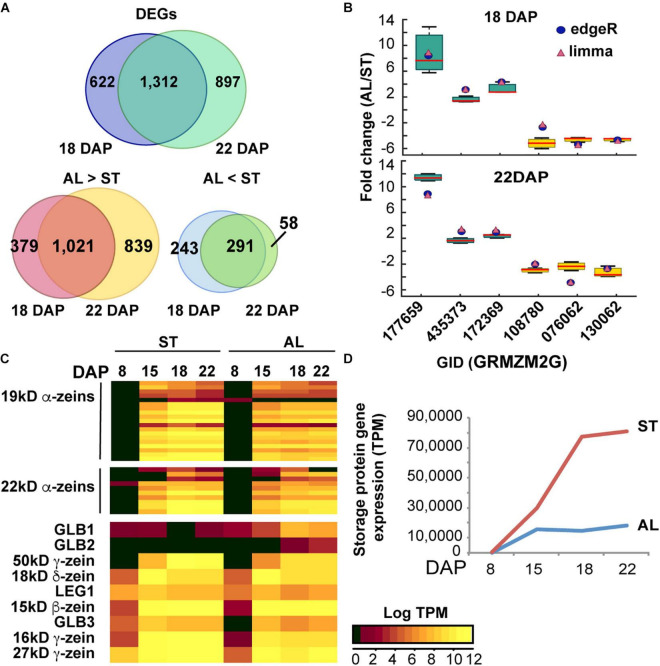
Gene expression analysis of maize endosperm. **(A)** Number of protein-coding genes expressed in aleurone (AL) and starchy endosperm (ST) at 18 and 22 DAP. **(B)** Differentially expressed genes (DEGs) in AL vs. ST at 18 and 22 DAP based on RNA-seq analysis. The Venn diagrams show the number of DEGs shared by the two developmental stages (18 and 22 DAP) or unique to either one or the other. **(C)** Quantitative RT-PCR of transcripts expressed specifically in either AL or ST endosperm. Fold changes measured by RT-qPCR are shown by Whisker box plots, and fold changes calculated by edgeR and limma are shown as blue circles and pink triangles, respectively. Expression was normalized to that of *UBC9.* Data is from three biological replicates. **(D)** Heat map and hierarchical clustering analysis of expression of genes coding for prolamins (zeins) and non-prolamin storage proteins. Log-transformed TPM values of expression levels are colored according to intensities (see color scale in figure). **(D)** Abundance of storage protein transcripts in aleurone (AL) and starchy endosperm (ST) during development.

We then searched for differentially expressed genes (DEGs) between aleurone and starchy endosperm samples by the edgeR and limma computational tools which generated consensus DEG datasets using a FDR < 0.05 and a fold change > 2. In total, we identified 1,934 and 2,209 DEGs when comparing aleurone vs. starchy endosperm samples at 18 and 22 DAP, respectively (Figure 4A). Consistent with more genes being expressed in aleurone than in starchy endosperm, 1,400 and 1,860 genes were preferentially expressed in aleurone and 534 and 349 were preferentially expressed in starchy endosperm at 18 and 22 DAP, respectively (Supplementary Table 3). The majority of DEGs at 18 and 22 DAP overlapped (Figure 4A), which suggests that few changes in mRNA accumulation occur between two time points. To verify the accuracy of our gene differential expression analyses, six DEGs (three preferentially expressed in aleurone and three preferentially expressed in starchy endosperm) with dissimilar predicted fold changes were tested by RT-qPCR analysis. As shown in Figure 4B, the fold changes calculated from RNA-seq data compared favorably to those measured by RT-qPCR for all six genes.

To infer cellular localizations and functions of the proteins encoded by these DEGs, we performed a gene ontology (GO) enrichment analysis (Supplementary Figure 2A). GO terms associated with the vacuolar membrane, lipid storage body, and ER were enriched in the DEGs preferentially expressed in aleurone cells, while GO terms associated with amyloplast, starch grain, and rough ER were enriched in the DEGs preferentially expressed in starchy endosperm cells (Supplementary Figure 2A), which agreed with the predominant functions of these tissues.

### Expression Patterns of Genes Connected to Storage Protein Synthesis and Accumulation

Given the differences in storage protein trafficking in aleurone vs. starchy endosperm tissues, we then examined whether transcript accumulation patterns of genes encoding storage proteins and/or proteins associated with ER protein bodies would be also different in the two tissues. To enhance the analysis, we interrogated gene expression in a wider developmental window by combining our transcriptome results at 18 and 22 DAP with other published RNA-seq datasets from maize endosperm at 8 and 15 DAP (Yi et al., 2015; Zhan et al., 2015). First, we analyzed the expression pattern of genes encoding storage protein (prolamins and non-prolamins) (Woo et al., 2001; Figures 4C,D and Supplementary Table 4). In the starchy endosperm, only ∼0.3% of the total transcripts at 8 DAP encoded storage proteins but this abundance progressively increased to 29, 77, and 81% at 15, 18, and 22 DAP, respectively (Figure 4D). Prolamin transcripts were most abundant and consistently represented 99% of the total starchy endosperm storage protein mRNAs in the 15 to 22 DAP developmental window. By contrast, transcripts for storage proteins in aleurone cells represented 0.02% of the total transcriptome by 8 DAP and increased modestly to 14 and 18% by 18 and 22 DAP, respectively (Figure 4D). Although prolamin and non-prolamin transcripts were equally represented at 8 DAP in aleurone cells, over 98% of the aleurone storage protein transcripts between 15 and 22 DAP encoded prolamin family members (Supplementary Table 4). Based on DEG analysis, all prolamin transcripts were preferentially expressed in starchy endosperm at 18 and 22 DAP (Supplementary Table 4). Pearson correlation coefficients (PCC) using log-TPM of all prolamin-encoding genes for aleurone vs. starchy endosperm at 8, 15, 18, and 22 DAP, respectively, were invariably high (0.87–0.99) for all four developmental stages, indicating a highly correlation for the same prolamin-encoding genes expressed in aleurone and starchy endosperm cells.

We then queried the datasets for transcripts encoding proteins shown previously to participate in ER protein body formation (Hunter et al., 2002; Holding et al., 2007; Yao et al., 2016), and/or found by proteomic studies (Wang et al., 2016) to associate with maize protein bodies, based on the rationale that critical factors needed to stabilize ER protein bodies and avoid their sequestration into vacuoles would be selectively expressed in starchy endosperm but not in aleurone cells. Among genes encoding known protein body-associated factors, we focused on those that were preferentially expressed in starchy endosperm at 18 and 22 DAP as defined by a Compartment Correlation (CS) Score $CS\left(starchyendosperm\right)score\;GeneA=\frac{\mathrm{sum}\left(\mathrm{all}\mathrm{TPM}\mathrm{of}\mathrm{GeneA}\mathrm{in}\mathrm{starchy}\mathrm{endosperm}\right)}{\mathrm{sum}\left(\mathrm{all}\mathrm{TPM}\mathrm{of}\mathrm{GeneA}\mathrm{in}\mathrm{starchy}\mathrm{endosperm}\mathrm{and}\mathrm{aleurone}\right)}$. For those protein-body associated factors preferentially expressed in starchy endosperm, a catalog with a CS (starchy endosperm) score >0.5 was generated, including five genes encoding RNA-binding proteins (RBP) which might participate in the trafficking of prolamin mRNAs to the ER (Crofts et al., 2004; Yang et al., 2014; Wang et al., 2016), eight genes encoding ER-resident chaperones (Wang et al., 2016), which might be required for stable accumulation of prolamins in the ER, 10 genes encoding proteins involved in stress response (Wang et al., 2016), 33 genes encoding proteins of various function (Wang et al., 2016), and four genes involved in protein body assembly [OP10 (Yao et al., 2016), FL1 (Holding et al., 2007), a FL1-like gene, and OP1; Hunter et al., 2002; Supplementary Table 5 and Supplementary Figures 2B–F]. However, similar to storage protein genes, the transcript abundances of protein body-associated factors in aleurone and starchy endosperm positively correlated [at all four developmental stages (PCC = 0.88–0.99)].

After examining DEGs between aleurone and starchy endosperm, particularly those involved in storage protein synthesis and those know or potentially involved in protein body formation, we did not find significant difference upon comparing the two tissues at 18 and 22 DAP. This suggests that microautophagy is not controlled by the differential expression of genes involved in storage protein synthesis and protein body formation.

### Identification of Proteins Associated With Maize Aleurone Tonoplast

Because the storage protein inclusions in aleurone vacuoles appeared to be directly engulfed by the vacuole *via* microautophagy (Figure 2), we reasoned that proteins responsible for this selectivity could be identified by proteomic analysis of the tonoplast. Toward this goal, we first isolated the microsomal and then the tonoplast fractions (Schumaker and Sze, 1985) of aleurone cells from 20 DAP seeds and performed co-immunoprecipitation (co-IP) with antibodies against H^+^-pyrophosphatases (H^+^-PPases), which are known to be enriched in the plant tonoplast, using the pre-immune serum as a negative control (Supplementary Figure 3). These antibodies were generated against a peptide from the PPi binding domain of *Arabidopsis* AVP1 (Paez-Valencia et al., 2011) that is fully conserved in their maize orthologs.

**FIGURE 5:**
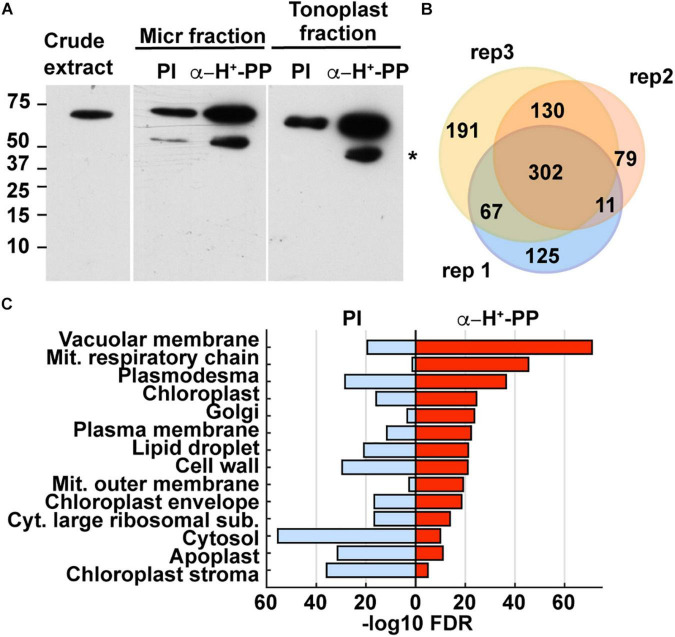
Identification of maize aleurone tonoplast proteins enriched by co-immunoprecipitation (co-IP) with anti-H+-PPase antibodies as shown by immunoblot and proteomic analyses. **(A)** Immunoblot detection of H+-PPases in maize aleurone crude extract, and immune-precipitated microsomal and tonoplast fractions with either pre-immune serum (PI; negative control) or anti-H+-PPase antibodies. The lower molecular band indicated by an asterisk is likely a degradation product. **(B)** Venn diagram showing the overlap of proteins detected by MS analysis in the three biological replicates immunoprecipitated with anti-H+-PPase antibodies. **(C)** Enriched GO terms of proteins from aleurone tonoplast fraction immunoprecipitated with either pre-immune serum (negative control) or anti-H+-PPase antibodies. The GO terms are ranked from top to bottom by their FDR values.

We analyzed the co-IPs by SDS-PAGE and immunoblot analysis with the anti- H^+^-PPase antibodies and found that the tonoplast fractions had the highest enrichment for H^+^-PPases (Figure 5A). These co-immunoprecipitates were then trypsinized and analyzed for protein content by tandem mass spectrometry (MS), using the MS1 scans of three biological replicates (analyzed by two technical replicants) for a semiquantitative measure of protein abundance. A total of 881 proteins where identified in the samples co-immunoprecipitated with the anti-H^+^-PPase antibodies, with most of them (72–85%) represented in two or more biological replicates (Figure 5B, Supplementary Figure 3, and Supplementary Tables 6,7), indicating good reproducibility of membrane fractionation and protein detection. Only 13–35% of these proteins were also detected in the pre-immune samples, indicating that non-specific binding to the beads was modest (Supplementary Figure 3 and Supplementary Tables 5,6).

Based on a gene ontology (GO) analysis, we found enrichment of predicted vacuole and tonoplast proteins in co-IP samples obtained with the anti-H^+^-PPase antibodies as compared to the pre-immune control which was most highly enriched in cellular constituents from the cytosol, chloroplast stroma, apoplast, cell wall, and cytosolic ribosomes (Figure 5C). To verify this enrichment, we performed a BLAST analysis to identify likely maize homologs for proteins present in *Arabidopsis* tonoplast proteome datasets generated from leaves (Jaquinod et al., 2007). We found 166 putative homologs of *Arabidopsis* tonoplast proteins in our aleurone tonoplast proteome, including four isoforms of inorganic H^+^-PPases and nine vacuolar ATP synthase subunits (Supplementary Table 7). Whereas only 2% of the 39,465 protein-coding genes in maize are predicted to encode tonoplast proteins, ∼18% of the proteins detected in our tonoplast samples were predicted, thus reflecting a ∼14 fold enrichment as calculated by the Fisher’s Exact test (*p-*value < 2.2e-16).

We next compared the protein abundance in the anti- H^+^-PPase co-immunoprecipitates to their corresponding negative controls to eliminate non-specifically bound proteins. This comparison identified 489 proteins enriched with “high” confidence (present in at least two biological replicates with of ≥2-fold increase compared to the corresponding negative controls), with another 265 proteins enriched with “medium” confidence (present in only one biological replicate, but not in the negative controls), and with the remaining 127 proteins designated as “low” confidence. To test the stringency of our selection thresholds, we again performed GO enrichment analysis for the three confidence groups. Proteins in the low confidence group were mostly associated with an assortment of cellular features (*e.g*., plasmodesma, cytosolic large ribosomal subunits, apoplast, cytosol, and cell wall), whereas proteins in the high- and medium- confidence groups combined included proteins largely associated with the tonoplast/vacuolar membrane (Supplementary Figure 4A). Based on the GO enrichment results, we concentrated our analyses on the 754 proteins with high/medium confidence.

Transcript abundances of these selected proteins in aleurone and starchy endosperm cells, from 8 to 22 DAP, showed their preferential expression in aleurone cells during storage protein accumulation. Among the 754 proteins, there were 155 preferentially expressed in the aleurone and 44 preferentially expressed in the starchy endosperm, respectively, at 18 and/or 22 DAP (Supplementary Table 7). The average and median CS scores for the high and medium confidence group was 0.69 and 0.67, respectively, for aleurone between 8 and 22 DAP while the PCC values were 0.88 and 0.94 at 8 and 15 DAP, respectively, but decreased to 0.63 at 18 and 22 DAP (Supplementary Figure 4B). Collectively, these results suggested that most proteins detected in our H^+^-PPase-enriched tonoplast fraction are preferentially expressed in aleurone cells during the delivery of storage proteins to their vacuoles, as compared to the starchy endosperm.

### Candidate Proteins With Potential Function(s) in Maize Aleurone Microautophagy

To identify candidate tonoplast-associated proteins connected to microautophagy, we focused on those from the high/medium conference groups with CS scores (AL) > 0.5 between 8 and 22 DAP showed (Supplementary Table 8), indicative of preferential expression in aleurone. We then manually verified for these 624 proteins, functional annotations related to membrane trafficking and remodeling based on public databases (MaizeGDB, Phytozome, UniProt, NCBI), which helped cull the list to 143 microautophagy candidates. This list included 15 proteins involved in lipid biosynthesis, 19 proteins involved in lipid modification, degradation, and binding, 36 proteins involved in vacuolar and endosomal trafficking, seven involved in membrane binding or modifying processes, 10 glycan-binding/lectin-type proteins, 20 involved in stress response and autophagy, and 41 of unknown function (Supplementary Figure 4C and Supplementary Table 8).

To further narrow down our list for subsequent localization studies, we considered their expression patterns and protein abundance in endosperm cell types based on our own RNA-seq/proteomic analyses and the maize proteome atlas (Walley et al., 2016), as well as their orthology to factors previously connected to microautophagy in other organisms. Among the 15 proteins involved in lipid biosynthesis, six are annotated as putative long chain acyl-CoA synthetases (Supplementary Table 8) that direct the incorporation of long-chain or very-long-chain fatty acids into phospholipids, which are major components of plant cell membranes (Shockey et al., 2002). Interestingly, long chain acyl-CoA synthetases are essential to the metabolism of sphingolipids (Ohkuni et al., 2013), which are a critical components of lipid rafts or membrane microdomains in mammals, and linked to sphingolipid-enriched microdomains in yeast vacuoles connected to the microautophagy of lipid droplets (Tsuji et al., 2017). We selected the long chain acyl-CoA synthetase encoded by GRMZM2G079236/Zm00001d00519 for further study because it showed relatively high mRNA and protein abundances in the endosperm.

Within the 19 proteins predicted to be involved in lipid modification, degradation, and binding, five had predicted lipase activity and five had at least one Ca^2+^-dependent lipid binding C2 (protein kinase C conserved region 2) domain (Supplementary Table 8). Phospholipases control membrane phospholipid composition, which then impacts membrane biophysical properties, shape, and recruitment of effector proteins (Rocha et al., 2014). C2 domains often mediate the membrane docking of cytosolic proteins, such as some phospholipases, small GTPases, vesicular trafficking proteins, membrane-repair proteins, or additional lipid-association capacity to integral proteins such as synaptotagmins (Corbalan-Garcia and Gómez-Fernández, 2014). The most important role of C2 domains is to activate membrane-associated protein modules in a Ca^2+^-dependent manner. Therefore, from this group, we selected GRMZM2G442551/Zm00001d046508, a putative phospholipase D alpha (annotated as phospholipase Dα5 in maize) with three C2 domains, GRMZM2G100864/Zm00001d036801, a predicted Ca^2+^-dependent lipid-binding phosphoribosyltransferase family protein with two C2 domains and whose putative *Arabidopsis* homologs, FTIP3 and 4 (FT INTERACTING PROOTEIN 3 and 4), are required for intracellular protein trafficking in meristematic tissues (Liu et al., 2018), and GRMZM2G050193/Zm00001d006238, which is a synaptotagmin-like protein and therefore potentially able to bind phospholipids, induce membrane curvature, and mediate contact sites between organelles (Ullah et al., 2021).

Among the 36 proteins involved in vacuolar and endosomal trafficking, the two dominant groups were predicted small GTPases or their regulators (10 proteins), and SNAREs and tethering factors (8 proteins; Supplementary Table 8). We chose one protein from each group. GRMZM2G158887/Zm00001d038709 is a predicted Rab7 GTPase similar to *Arabidopsis* RABG3F, mammalian Rab7, and yeast Ypt7, which are involved in transport to the lysosome/vacuole (Cantalupo et al., 2001; Cui et al., 2014) and in yeast piecemeal microautophagy of the nucleus (Krick et al., 2008). GRMZM2G330772/Zm00001d007902 is one of two putative SYP1 SNAREs found in our protein list and whose *Arabidopsis* homolog controls vesicle trafficking in pollen tubes (Slane et al., 2017).

Among the 10 glycan-binding proteins, eight were predicted lectins. In mammalian cells, cytosolic galectins can recognize luminal glycoprotein domains in damaged lysosomal membranes and mediate their repair (Radulovic et al., 2018), activity that could be required during microautophagy. Among the identified lectins, three were jacalin-related lectins, which in other species have been shown to participate in pathogen defense (Weidenbach et al., 2016; Esch and Schaffrath, 2017), and four were ricin B-like *Euonymus* lectin (EUL) lectins, with a single orthologue in *Arabidopsis*, EULS3, mediating stomatal closure (Van Hove et al., 2015) and abscisic acid signaling in roots (Dubiel et al., 2020). In maize, there are eight jacalin-like genes and five genes coding for EUL-related lectins, four of which were enriched in the anti-H^+^-PPase co-immunoprecipitates from aleurone tonoplast. We selected one of the putative EUL-like lectins, GRMZM2G120304/Zm00001d040190, for further analysis.

Within the 20 proteins involved in stress-response and autophagy-related processes, we were surprised to see the two paralogues of BCL-2-associated athanogene 7 (BAG7), GRMZM2G472346/Zm00001d014946 and GRMZM2G041765/Zm00001d45596. Mammalian BAG proteins regulate autophagy and apoptosis and their plant counterparts appear to have more diverse functions. *Arabidopsis* BAG7 is involved in unfolded protein response and apoptosis and localize to the ER lumen (Williams et al., 2010) whereas BAG6 localizes to vacuoles and induces autophagy and resistance against fungal pathogens (Li et al., 2016). The two maize BAG7 paralogues are only moderately similar to *Arabidopsis* BAG7 (Supplementary Table 9) and lack a signal peptide, suggesting that maize BAG7 has different subcellular localizations and function. We selected GRMZM2G472346/Zm00001d014946 for further analysis. Additionally, two proteins of unknown function were added to the candidate list, GRMZM2G158788/Zm00001d009718 and GRMZM2G305851/Zm00001d038085, both of which are preferentially expressed in maize aleurone at 18 and/or 22 DAP and have high transcript accumulation and protein abundance in aleurone.

### Localization and Tonoplast Remodeling Capabilities of Candidate Proteins Overexpressed in *Arabidopsis* Protoplasts

**FIGURE 6:**
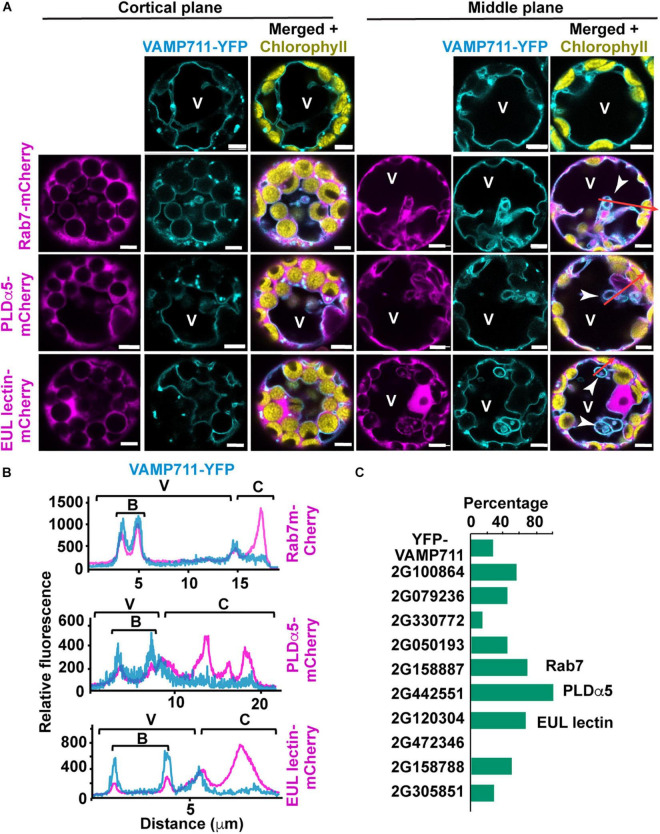
Expression of microautophagy candidate protein in *Arabidopsis* leaf protoplasts. Protoplasts were isolated from leaves of transgenic plants expressing VAMP711-YFP and transformed with microautophagy candidate genes tagged with m-Cherry. **(A)** Representative images of cortical and middle plane views of protoplasts co-expressing VAMP711-YFP and either Rab7-, PLDα5-, or EUL lectin- mCherry fusions. Tonoplast invagination are highlighted by white arrowheads. **(B)** Fluorescence intensity profiles of VAMP711-YFP and the three candidate proteins fused to mCherry along the red arrows **(A)**. These areas include vacuolar (V), bulb (B), and cytoplasmic (C) regions. **(C)** Percentage of protoplasts with rounded tonoplast invaginations or “bulbs” (*n* = 7–14 protoplasts for each combination). Chart shows results from one representative experiment. V, vacuole. Scale bars = 5 μm.

The 10 candidate proteins described above, including four proteins with predicted function in membrane lipid metabolism or binding, three with membrane binding/remodeling/trafficking capabilities, one with putative function in stress-response or autophagy (Supplementary Table 8), were then tested for their ability to associate with and remodel the tonoplast when expressed as mCherry translational fusions in *Arabidopsis* leaf protoplasts stably expressing the tonoplast marker VAMP711-YFP (Geldner et al., 2009). Expression of BAG7-mCherry surprisingly induced protoplast death and was not analyzed further. For the remainder, we imaged cortical and middle planes of randomly selected protoplasts using laser confocal microscopy to determine the subcellular localization. While their subcellular distributions differed, they were all cytoplasmic and in contact with the tonoplast (Figures 6A–C and Supplementary Figure 5).

We then examined the ability of these proteins to alter vacuole morphology by analyzing the tonoplast profiles of the transformed protoplasts. The expression of GRMZM2G158887 (Rab7), GRMZM2G442551 (PLDα5), and GRMZM2G120304 (EUL-lectin) caused a 3-fold or greater increase in the number of rounded tonoplast invaginations into the vacuolar lumen [also known as “bulbs” (Saito et al., 2002; Han et al., 2015)], compared to the protoplasts expressing VAMP711-YFP alone (Figure 6C).

To confirm the association of the mCherry-tagged proteins with the tonoplast, we generated fluorescent intensity profiles along regions of protoplasts that encompassed the vacuole, bulbs (when present), and cytoplasm; here, we found that Rab7, PLDα5, and the EUL lectin consistently associated with the tonoplast and bulbs (Figure 6B). The ability of these three proteins to promote changes in vacuolar morphology is consistent with a role in microautophagy and therefore, we decided to further characterize the subcellular localizations of Rab7, PLDα5, and the EUL-lectin in more detail *via* biolistic bombardment of *in vitro* cultured maize endosperms with mCherry tagged versions (Gruis et al., 2006).

### PLDα5 and an *Euonymus* Lectin-Lectin Are Enriched in Tonoplast Domain Involved in Microautophagy of Storage Proteins in Aleurone Cells

From developing Hi-II kernels at 8 DAP, we isolated the endosperms, and incubated them on solid-medium plates for 3–5 days *in vitro* (DIV) at 28°C (Gruis et al., 2006), which induced the successful *in vitro* differentiation of aleurone and starchy endosperm cells within a few days (Gruis et al., 2006; Reyes et al., 2010). To determine the developmental window in which protein storage vacuoles engulfed protein bodies in aleurone cells, we stained *in vitro*-cultured endosperms with the vacuolar dye 2′,7′-bis(2-carboxyethyl)-5,6-carboxyfluorescein acetoxymethyl ester (BCECF-AM) (Brauer et al., 1995; Swanson and Jones, 1996). BCECF-AM diffuses across cellular membranes, and once its ester group is cleaved by intracellular esterases, its protonated form provides a fluorescent reporter for vacuoles (Swanson and Jones, 1996). As storage proteins are autofluorescent, most strongly when excited with near-UV light (405 nm), but much less when excited by 488 or 561 nm light (Supplementary Figure 6A). We used 405 nm excitation for detecting autofluorescence of storage proteins and 488 nm light to excite BCECF-AM. In maize aleurone cells, BCECF-AM labels the vacuolar lumen as well as the included storage protein (not those outside), allowing us to distinguish them from those protein bodies still located in the cytoplasm and therefore, unstained by BCECF-AM (Supplementary Figure 6B, arrowheads). At 8 DAP + 3–4 DIV, the aleurone vacuoles were 5–10 mm in maximum diameter, irregular in shape and size, and peripherally associated with cytoplasmic storage protein bodies (Supplementary Figure 6B), thus resembling vacuoles from *in-planta* differentiated aleurone cells at 15 DAP (Figure 2A). Upon further maturation (*i.e*., 8 DAP + 5 DIV) (Supplementary Figure 6C), these protein storage vacuoles became smaller (1–3 mm in diameter), more uniform in sizes, and with one or two large storage protein inclusions, similar to the vacuoles of aleurone cells differentiated *in planta* at 20–24 DAP (Figure 1). At this stage, no more protein bodies were detected in the cytoplasm, outside the protein storage vacuoles. This is consistent with our previous observations that aleurone and starchy endosperm differentiation progresses faster in *in vitro* culture than seen *in vivo* (Reyes et al., 2010).

Once we established that *in vitro* cultured endosperms differentiate aleurone cells that perform microautophagy of storage protein bodies, we transformed cultured endosperm (8 DAP + 2 DIV) by biolistic bombardment to express mCherry translational fusions of the previously selected microautophagy candidate proteins, together with the maize tonoplast marker TIP1-YFP driven by its native promoter (Krishnakumar et al., 2015). However, the TIP1-YFP protein failed to reach the tonoplast in aleurone cells and was instead retained in the ER, as seen by its strong signal overlap with that seen with the mCherry-KDEL ER marker (Supplementary Figure 7A). Therefore, we used BCECF-AM staining and bright field images to detect aleurone vacuoles and observed the localization of Rab7, PLDα5, and lectin-like fused to mCherry between 24 and 72 h after bombardment (*i.e.*, 8DAP +3–5DIV).

**FIGURE 7:**
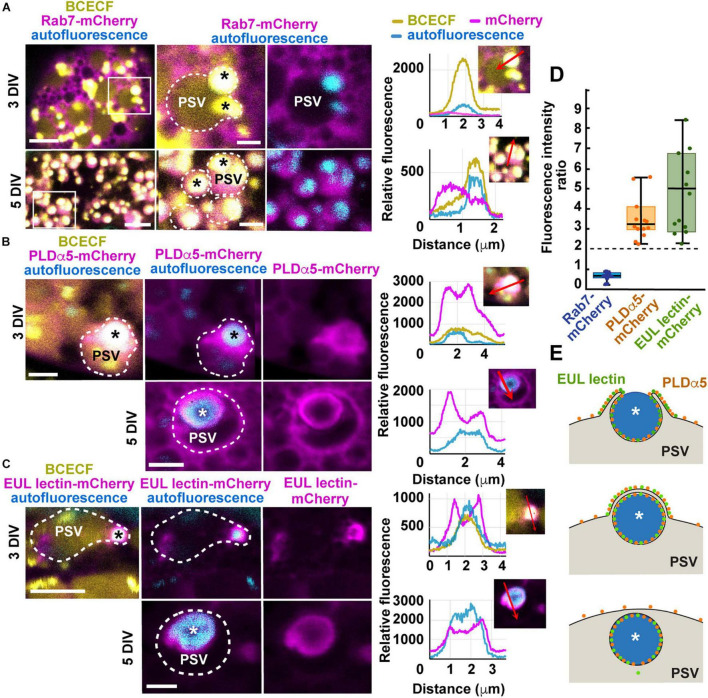
Expression of mCherry translational fusions of Rab7, PLDα5, and EUL-lectin protein in aleurone cells of *in vitro*-cultured endosperms transformed by biolistic bombardment. **(A–C)** Endosperms were collected at 8 DAP and placed on culture plates, bombarded 2 days later, and imaged between 24 h (8 DAP + 3 DIV) and 72 h after bombardment (8 DAP + 5 DIV). Protein storage vacuoles (PSVs) were detected by BCECF-AM staining and storage protein bodies (asterisks), by their intrinsic fluorescence. The outline of PSVs is indicated by dotted lines. **(A)** mRab7-mCherry shows a cytoplasmic distribution at 3 DIV but it is almost completely internalized into PSVs by 5 DIV. The areas highlighted by a white box in the panels on the left are shown at higher magnification in the middle and right panels. Fluorescence intensity profiles were calculated along the red arrows indicated in the selected panels. **(B)** PLDα5-mCherry localizes to the tonoplast of PSVs and around partially or completely engulfed protein bodies, at 3 DIV and 5 DIV, respectively. **(C)** EUL lectin-mCherry localizes almost exclusively to the tonoplast domain surrounding partially or completely engulfed protein bodies. **(D)** Boxplot showing fold enrichments of PLDα5-mCherry and EUL lectin-mCherry average fluorescence intensity around the storage protein inclusions compared to other areas on the PSV tonoplast. Only regions with no saturated pixels were measured (*n* = 14 PSVs from PLDα5-mCherry and 12 PSVs for EUL lectin-mCherry). The expected ratio (2) for proteins evenly distributed across the tonoplast is indicated by a dashed line. **(E)** Diagram showing the main steps in microautophagy of protein bodies in maize aleurone cells and the distribution of PLDα5 and the EUL lectin during this process. Scale bars = 5 μm.

Rab7-mCherry localized mainly to cytoplasm in young aleurone cells (8 DAP + 3 DIV), but it was almost exclusively found inside protein storage vacuoles by 5 DIV (Figure 7A). However, it is unclear whether that Rab7-mCherry was specifically internalized into vacuoles in aleurone cells since large part of the ER marker mCherry-KDEL signal also arose from the lumen of aleurone vacuoles (Supplementary Figure 7A). Which is consistent with the high macroautophagic activity reported in aleurone cells at this stage (Zhang et al., 2020).

PLDα5- and EUL-lectin-mCherry were both associated with the tonoplast at 3 DIV, most frequently with the tonoplast domain surrounding partially internalized protein bodies at the periphery of the vacuoles (Figures 7B,C). By 5 DIV, both PLDα5 and EUL-lectin fusion proteins could be detected around the storage protein inclusion now fully internalized into the vacuole. However, both at 3 and 5 DIV, lectin-like was almost exclusively found at the domain surrounding the protein body/storage protein inclusion while PLDα5 decorated the entirety of the tonoplast surface (Figures 7B,C).

To test whether the overexpression of PLDα5- and EUL-lectin-mCherry affects the vacuolar delivery of prolamins, we quantified the number of aleurone cells with cytoplasmic protein bodies in transformed and control endosperms. At 5 DIV, there were no protein bodies left in the cytoplasm of either control or transformed aleurone cells and storage protein aggregates were only be seen inside vacuoles (Supplementary Figure 6C). In average, at 3 DIV, only 10–17% of aleurone cells (control and transformed) showed cytoplasmic protein bodies (Supplementary Figure 8) whereas all vacuoles already contain storage protein aggregates, suggesting that at least the initiation of vacuolar sequestration of protein bodies is almost complete by 3 DIV. Thus, although we did not find statistically significant differences in the localization of protein bodies among transformed and control aleurone cells, it is difficult to establish based on this assay whether the overexpression of PLDα5- and EUL-lectin-mCherry could have any subtle effect on the trafficking of protein bodies.

Based on our electron microscopy, there should be two layers of tonoplast near the pair of engulfing flanks as their tips surround the protein body and anastomose during microautophagy (Figures 1D,E). To determine whether the stronger fluorescent signal of PLDα5- and EUL-lectin-mCherry around the protein body was merely due to the presence of two anastomosing tonoplast membranes in this region, we measured fluorescence intensity of PLDα5,- EUL- lectin-, and Rab7-mCherry (control) at the tonoplast domains associated with partially engulfed protein bodies and the tonoplast elsewhere and calculated the ratio. If the increase in signal was due to the presence of two closely located tonoplast membranes, this ratio should be close to two, as we showed previously for general tonoplast markers during the microautophagy of anthocyanin vacuolar inclusions (Chanoca et al., 2015). However, most of the tonoplast sites associated with microautophagy of storage proteins in aleurone cells showed more than two-fold increases in fluorescence intensity for both PLDα5- and EUL-lectin-mCherry (average and median fold increases were 3.2 and 4.1 for PLDα5-mCherry and 4.6 and 4.1 for EUL-lectin-mCherry, respectively) as shown in Figure 7D, which supports that PLDα5 and the EUL-lectin accumulate specifically at the tonoplast domains that mediate microautophagy of storage protein bodies (Figure 7E). Rab7-mCherry, which does not seem to associate with the tonoplast, show an intensity ratio close to 0.7. Thus, our study has uncovered two proteins, PLDα5 and the EUL-lectin, that localize preferentially to tonoplast domains promoting microautophagy in plants.

## Discussion

In this study, we have analyzed the process by which ER-derived storage protein bodies in maize aleurone cells are packaged in vacuoles *via* route whereby the bodies are directly engulfed by microautophagy (Figures 1, 2). Interestingly, similar storage proteins are exclusively retained in the ER of starchy endosperm cells as ER protein bodies. As shown by our previous studies, this autophagic delivery in maize aleurone cells does not rely on the core ATG machinery (Reyes et al., 2011; Li et al., 2015). The pathways responsible for either the retention of storage proteins in the ER of starchy endosperm cells or their delivery to vacuoles in aleurone cells seem to be an integral part of the differentiation programs of the two cell types. In fact, when young aleurone cells divide periclinally, the resulting sub-aleurone cell that ultimately will become part of the starchy endosperm transiently accumulate storage proteins in both ER bodies and microautophagic vacuolar inclusions (Figure 3). Our study also identified two proteins, PLDα5 and a EUL-lectin, that accumulate preferentially at the tonoplast domain engaged in microautophagy of ER protein bodies, revealing potentially novel factors in this poorly understood cellular process.

### Macroautophagy vs. Microautophagy

Microautophagy is poorly characterized in most organisms, especially in plants. Although both macro- and microautophagy results in the vacuolar deposition of a single-membrane-bound autophagic bodies containing cytoplasmic cargo, the mechanisms by which these structures are assembled are rather different. During macroautophagy, autophagosomes are assembled in the cytoplasm with the participation of ATG8 and the ATG8-conjugating machinery using the ER and other organelles as membrane sources (Le Bars et al., 2014; Zhuang et al., 2017). By contrast, microautophagy engages on the vacuolar membrane itself to surround and sequester cytoplasmic cargo. Consequently, autophagic bodies released into vacuoles by macroautopaghy are surrounded by a membrane derived from autophagosomes, while those generated by microautophagy are enclosed by the tonoplast.

Both macro- and micro-autophagy can be non-selective when mediating the bulk degradation of cellular components, or selective by using specific autophagic receptors to select individual organelles or macromolecular complexes as cargo (Mijaljica et al., 2011). In *Saccharomyces cerevisiae* cells undergoing diauxic shift (Oku et al., 2017) or cultured under nitrogen starvation conditions (Muller et al., 2000), long tubular invaginations of the vacuolar membrane facilitate the non-selective uptake of cytosolic contents by microautophagy. Specific cargo such as peroxisomes, mitochondria, nuclear fragments, lipid droplets, and endosomes in animal and yeast cells (Sakai et al., 1998; Nowikovsky et al., 2007; Kawamura et al., 2012) and anthocyanin aggregates and chloroplasts damaged by highlight in plant cells (Chanoca et al., 2015; Nakamura et al., 2018) appear to be delivered to vacuoles *via* selective microautophagy.

Whereas the core machinery regulating macroautophagy is well known and conserved in most eukaryotes, microautophagy has been documented only in a few cases and its underpinning molecular mechanisms seem to be more diverse (Schuck, 2020). For example, some microautophagy routes, including the microautophagy of photodamaged chloroplasts (Izumi et al., 2017; Nakamura et al., 2018), depend on the core ATG machinery (Muller et al., 2000; Uttenweiler et al., 2007; Krick et al., 2008) while others, such as diauxic shift-induced microautophagy in yeast (Oku et al., 2017) and microautophagy of anthocyanin-aggregates in *Arabidopsis*, do not required the ATG core pathway (Chanoca et al., 2015). In the absence this core pathway and it nexus ATG8, an intriguing question is how such microautophagy acheives selectivity given that ATG8 typically servers as the tether in macroautophagy that links receptors bearing specific targets to the enveloping autophagic membrane. Do the known ATG8-independent microautophagic routes employ alternative docking protein(s) along with their cognate receptors?

Some microautophagy processes in yeast, including ER-phagy and degradation of vacuolar membranes, depends on the ESCRT (Endosomal Sorting Complex Required for Transport) machinery (Oku et al., 2017; Morshed et al., 2020; Schäfer et al., 2020; Yang et al., 2020; He et al., 2021). During nutrient-rich conditions, TORC1 (Target of Rapamycin Complex 1) phosphorylates the ESCRT component Vps27 to antagonize with its function in microautophagy (Hatakeyama and De Virgilio, 2019). Vps27 is part of the ESCRT-0 complex that binds phosphoinositol-3-phosphate on membrane. Plants do not have obvious orthologs of Vps27 so whether a similar microautophagy pathway occurs in plants is presently unclear. However, we do know that the molecular mechanism controlling microautophagy of anthocyanin aggregates in plants seems to be independent of phosphatidylinositol 3-kinase activity (Chanoca et al., 2015). In support, we failed to detect any proteins belonging to the ESCRT complexes in our proteomic analysis of the maize aleurone tonoplast (Supplementary Tables 5,6).

Microautophagy has just begun to be characterized in plants, both at cellular and molecular levels (Sieńko et al., 2020). We previously reported that anthocyanin aggregates in *Arabidopsis* cotyledons and lisianthus (*Eustoma grandiorum*) petals are delivered to the vacuole by microautophagy. In this case, the tonoplast protrudes into the cytoplasm and becomes tightly associated with cytoplasmic anthocyanin aggregates. As the tonoplast completely wraps around the anthocyanin aggregates in a double-membrane cup-shaped structure, the distal tonoplast regions fused and closed, releasing the anthocyanin core (anthocyanin vacuolar inclusion) surrounded by a single tonoplast-derived membrane, which is by definition a microautophagic body. In other cases, such as microautophagy of photodamaged chloroplasts, the tonoplast invaginates without protrusions to sequester targeted chloroplasts. The vacuolar membrane deformation patterns seen during microautophagy of ER storage protein bodies includes both protrusions and invaginations, and resemble those of piecemeal microautophagy of the nucleus in carbon and nitrogen-starved *S. cerevisiae* cells. In this case, nuclear membrane protrusions establish tight contact sites with the vacuolar membrane. Once both organelles are in contact, the vacuolar membrane invaginates and closes around pinched-off portions of the nuclear envelope (Roberts et al., 2003). Once released into the vacuole, the surrounding membrane and the whole microautophagic body is degraded. Interestingly, as opposed to the reported cases of microautophagy in animals and yeast, microautophagy of anthocyanin aggregates and storage protein bodies seen in plants have storage functions rather than breakdown/recycling.

The cellular and environmental factors that activate microautophagy in plants are unknown. It is interesting, however, to note that in three documented cases of plant microautophagy, *i.e.*, sequestration of anthocyanin aggregates (Chanoca et al., 2015), photodamaged chloroplasts (Izumi et al., 2017; Nakamura et al., 2018), and ER storage protein bodies (this study), the cargo could be sensed as potentially toxic by the cell. Prolamins evolved in grasses as the dominant type of storage proteins in the endosperm. It is assumed that they derived from genes encoding vacuolar storage proteins but harbor mutations leading to the introduction of cysteines and hydrophobic residues that promote proteins aggregation and concomitant retention within the ER (Pedrazzini et al., 2016). Alternatively, it is unclear how such a massive accumulation of aggregated proteins within the ER does not cause deleterious effects and ER stress in starchy endosperm cells. This could be connected to the fact that the starchy endosperm undergoes programmed cell death and therefore possibly able to tolerate protein aggregation without triggering ER stress. Instead, aleurone cells remain alive during seed maturation and germination and thus might need microautophagy to sequester prolamins aggregates away from the ER and thus avoid proteoxic stress.

Protein aggregation alone does not necessarily imply ER retention. The stable accumulation and retention of protein bodies within the ER require controlled prolamin stoichiometry (Guo et al., 2013) and the action of a number of factors, including ER chaperones and ER membrane proteins (Holding et al., 2007; Pedrazzini et al., 2016). We found that although transcripts of prolamins and protein body-organization factors are much more abundant in the starchy endosperm than in aleurone cells, their relative proportions and time of expression were comparable in both cell types. It is possible that the expression of factors needed for the retention of prolamins in the ER must exceed certain threshold for ER protein bodies to accumulate, a situation that is never reached in aleurone cells. Alternatively, both endosperm cell types could be capable of forming ER protein bodies but the pathway that mediates microautophagy of prolamin aggregates is active only in aleurone cells.

### Molecular Characterization of Microautophagy

Vacuolar dynamics in plants is controlled by hormones (*e.g.*, auxin, abscisic acid) and a large array of trafficking factors, including SNAREs, Rab GTPases, tethering factors, membrane lipids, and phospholipases (Saito et al., 2011; Löfke et al., 2015; Brillada et al., 2018; Takemoto et al., 2018). Whether any of these factors influence microautophagy is currently unknown. Among the 10 candidate proteins selected here that were enriched in the tonoplast of aleurone vacuoles at 20 DAP, three (a Rab7 GTPase, PLDα5, and an EUL lectin) induced tonoplast invaginations when overexpressed in leaf protoplasts. Whereas Rab7 mostly localized to the cytoplasm, PLDα5 and the EUL lectin preferentially localized to the tonoplast regions wrapping around the storage protein bodies in developing aleurone cells (Figures 7A–C), consistent with a role in microautophagy.

PLDs catalyzes the hydrolysis of glycerol-phospholipids to produce phosphatidic acid and water-soluble head groups. In yeast, mammals, and plants, PLDs mainly act on phosphatidylcholine (Rudge et al., 2001; Zien et al., 2001), but plant PLDs can also hydrolyze phosphatidylethanolamine and phosphatidylglycerol (Pappan et al., 1998). All yeast and mammal PLDs have a Phox homology (PX) domain followed by an N-terminal pleckstrin homology (PH) domain, which can bind to different phospholipids including phosphoinositides. Instead, both maize PLDα5 and its closest *Arabidopsis* homology PLDa1, just like most plant PLDs, have a C2 domain in the N-terminus (Chen et al., 2017). The lipid-binding specificity and catalytic activity of plant PLDs with C2 domains are regulated in a Ca^2+^-dependent manner. In *Arabidopsis*, Ca^2+^ is needed for the binding of the PLD C2 domain to phosphatidylcholine (Zheng et al., 2000) and in maize, Ca^2+^ concentration regulates differently catalytic activities of the soluble and membrane-bound PLD pools (Brauer et al., 1991). Whereas the critical role of PLDs and phosphatidic acid in cell signaling, abscisic acid-related, and stress responses has been widely documented in plants (Zhang et al., 2004, 2009; Zhao, 2015; Yang et al., 2021), their roles in membrane remodeling are less clear. *Arabidopsis* PLDζ2, which has a PX and a PH domain at the N-terminus, localizes to the tonoplast, induces tonoplast invaginations under phosphate deprivation, becoming more enriched in those tonoplast invaginations (Yamaryo et al., 2008). PLD could play a role in tonoplast membrane remodeling in two major ways, either by promoting negative membrane bending (away from the cytoplasm) facilitated by the cone shape of phosphatidic acid, and/or by controlling the recruitment of phosphatidic acid -binding effectors, such as trafficking factors (Lam et al., 2008), the kinase TOR, a master regulator of both macro- and microautophagy (Kvam and Goldfarb, 2007; Toschi et al., 2009; Hatakeyama and De Virgilio, 2019).

The lectin identified in this study belongs to the plant EUL family. Through their unique abilities to recognize different glycan ligands, lectins are involved in ER protein quality control and sorting of glycoproteins for secretion, sensing both pathogens on the cell surface, and membrane damage (Van Damme et al., 2004; Tsaneva and Van Damme, 2020). Although a large group of lectins, including the EULs, localize to the cytoplasm, their potential association with the tonoplast and a role in membrane remodeling have not been explored. However, mammalian galectins can induce plasma membrane invaginations and recognize lumenal glycans in damaged lysosomes to mediate either their repair (Jia et al., 2020) or autophagic clearance (Chauhan et al., 2016). Galectins have no obvious homologs in plants but their roles in membrane binding and remodeling depend largely on their abilities to bind specific glycans associated with membranes and to recruit other proteins involved in membrane remodeling or autophagy, functions that could be played by the cytoplasmic plant lectins. Interestingly, we found that four of the five maize EUL lectins were preferentially expressed in aleurone cells and enriched in the aleurone tonoplast fraction. One could hypothesize that lectins associate with the tonoplast as result of vacuolar damage and exposure of lumenal glycans during subcellular fractionation. However, both the ability of the EUL lectin to modify vacuolar morphology in *Arabidopsis* protoplasts and to localize specifically to tonoplast domains engaged in microautophagy of protein bodies in aleurone cells argues against this possibility. Interestingly, the only *Arabidopsis* EUL lectin, AtEULS3, is involved in stomatal closure in response to abscisic acid. Although the exact mechanism of AtEULS3 action is not known, it is tempting to speculate that it could control vacuolar remodeling, which is required for guard cell expansion/contraction and subsequent stomatal opening/closing. In fact, large vacuolar invagination could provide a reserve of tonoplast membrane in closed guard cells (Tanaka et al., 2007).

Future studies using mutants for PLDα5 and EUL lectins will be needed to confirm their role in plant microautophagy. In addition, the analysis of more candidate proteins identified in this study could reveal additional factors controlling microautophagy in aleurone cells and potentially in other cell types and species.

## Data Availability Statement

The raw sequence, msf, and xml files for the mass spectrometry data sets are available at the ProteomeXchange database under the accession number PXD011039 within the PRIDE repository.

## Author Contributions

XD, XZ, JP-V, FM, FR, and KM conducted the experiments and analyzed the data. EG, RV, and MO together with the other co-authors, designed the experiments and analyzed the results. XD and MO wrote the manuscript with input from all authors. All authors have read and approved the final version of the manuscript.

## Conflict of Interest

The authors declare that the research was conducted in the absence of any commercial or financial relationships that could be construed as a potential conflict of interest.

## Publisher’s Note

All claims expressed in this article are solely those of the authors and do not necessarily represent those of their affiliated organizations, or those of the publisher, the editors and the reviewers. Any product that may be evaluated in this article, or claim that may be made by its manufacturer, is not guaranteed or endorsed by the publisher.

## Footnotes

1
https://www.bioinformatics.babraham.ac.uk/projects/fastqc/

2
http://github.com/jdstorey/qvalue

3
http://www.hartfaalcentrum.nl/index.php?main=files&sub=LinRegPCR

4
www.maizegdb.org

5
ftp://ftp.gramene.org/pub/gramene/archives/PAST_RELEASES/release51/

## Abbreviations

ATG: autophagy-related
DAP: days after pollination
DEG: differentially expressed gene
ER: endoplasmic reticulum
FDR: false discovery rates
H^+^-PPase: H^+^-pyrophosphatase
logFC: log2 fold changes
log-TPM: log-transformed transcript per million (TPM)
PCC: Pearson Correlation Coefficient
PSV: protein storage vacuole
RT-qPCR: reverse transcription quantitative polymerase chain reaction.
