# Supplementary material for: Microautophagy Mediates Vacuolar Delivery of Storage Proteins in Maize Aleurone Cells
Source: Front Plant Sci. 2022 Feb 18;13:833612. doi: 10.3389/fpls.2022.833612 (PMC8894768; doi:10.3389/fpls.2022.833612)
Supplement: Supplementary file 2 [file Data_Sheet_2.docx]

**
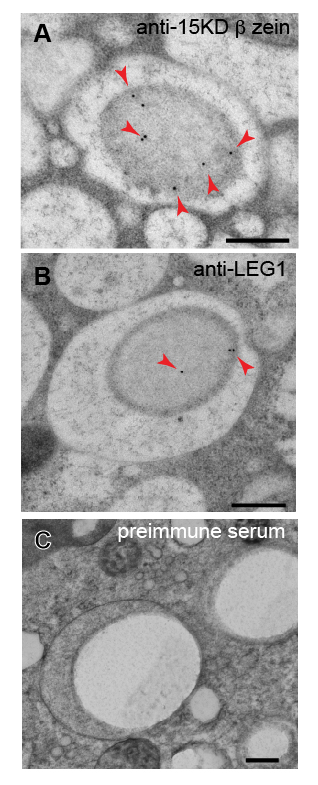
Supplemental Figures**

**Supplemental Figure S1**. Immunolocalization of proteins in aleurone vacuoles. (A) Anti-15kD β-zein labeling on storage protein inclusion (arrowheads). (B) Anti-legumin 1 (LEG1) labeling on storage protein inclusion (arrowheads). (C) Pre-immune serum control for the labeling of H^+^ pyrophosphatases shown in Fig. 1G and H. Scale bars: 400 nm.

**
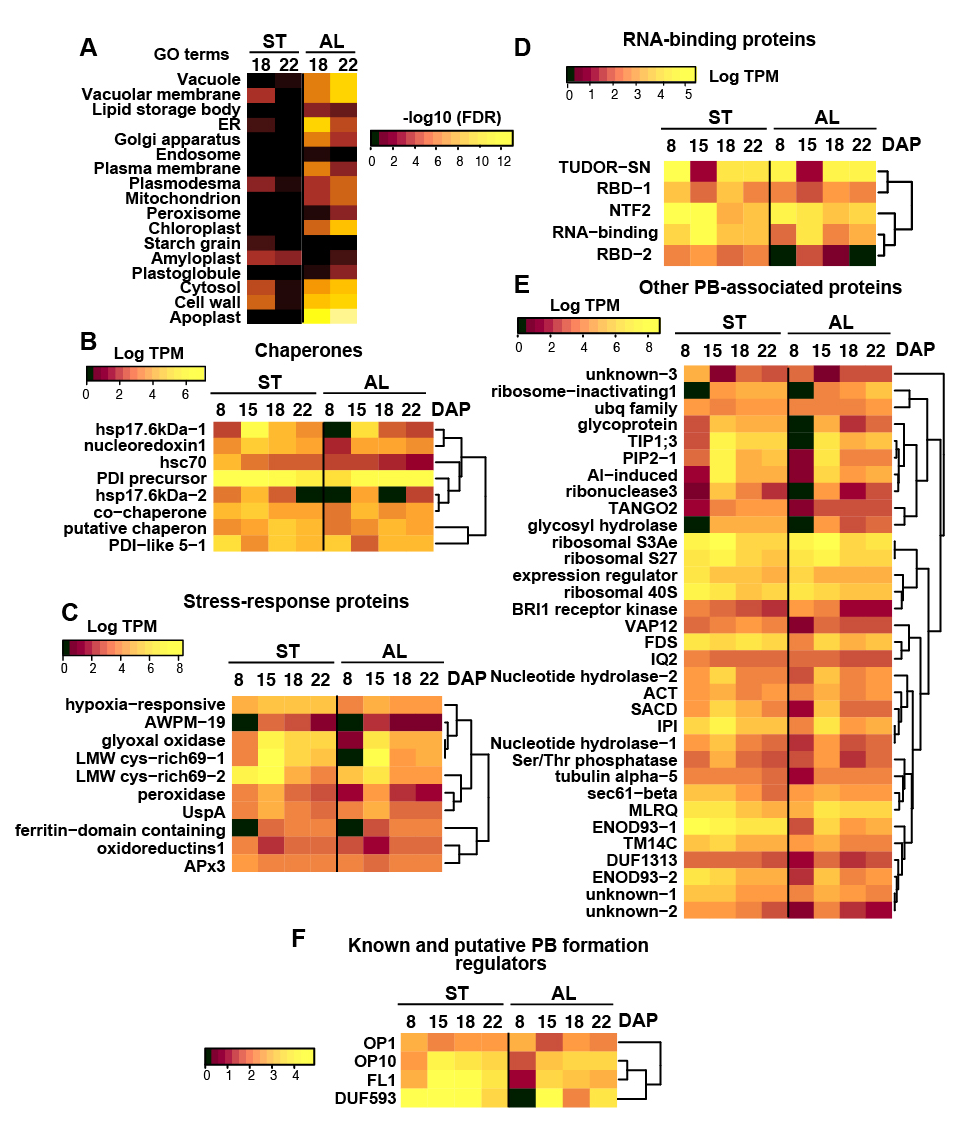
**

**Supplemental Figure S2.** Heat map diagram and hierarchical clustering analysis of expression of genes involved in protein body assembly**.** Log-TPM values are depicted according to color scale.

**
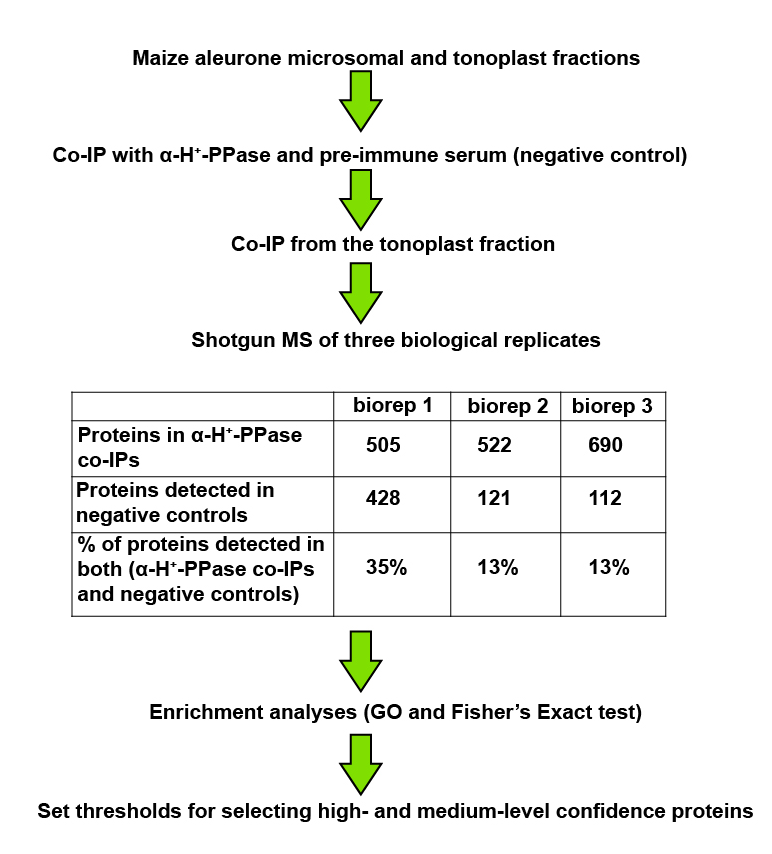
**

**Supplemental Figure S3.** Steps in the fractionation, co-immunoprecipitation (co-IP), mass spectrometry (MS), and GO term enrichment analysis to identify tonoplast associated proteins in aleurone cells.

**
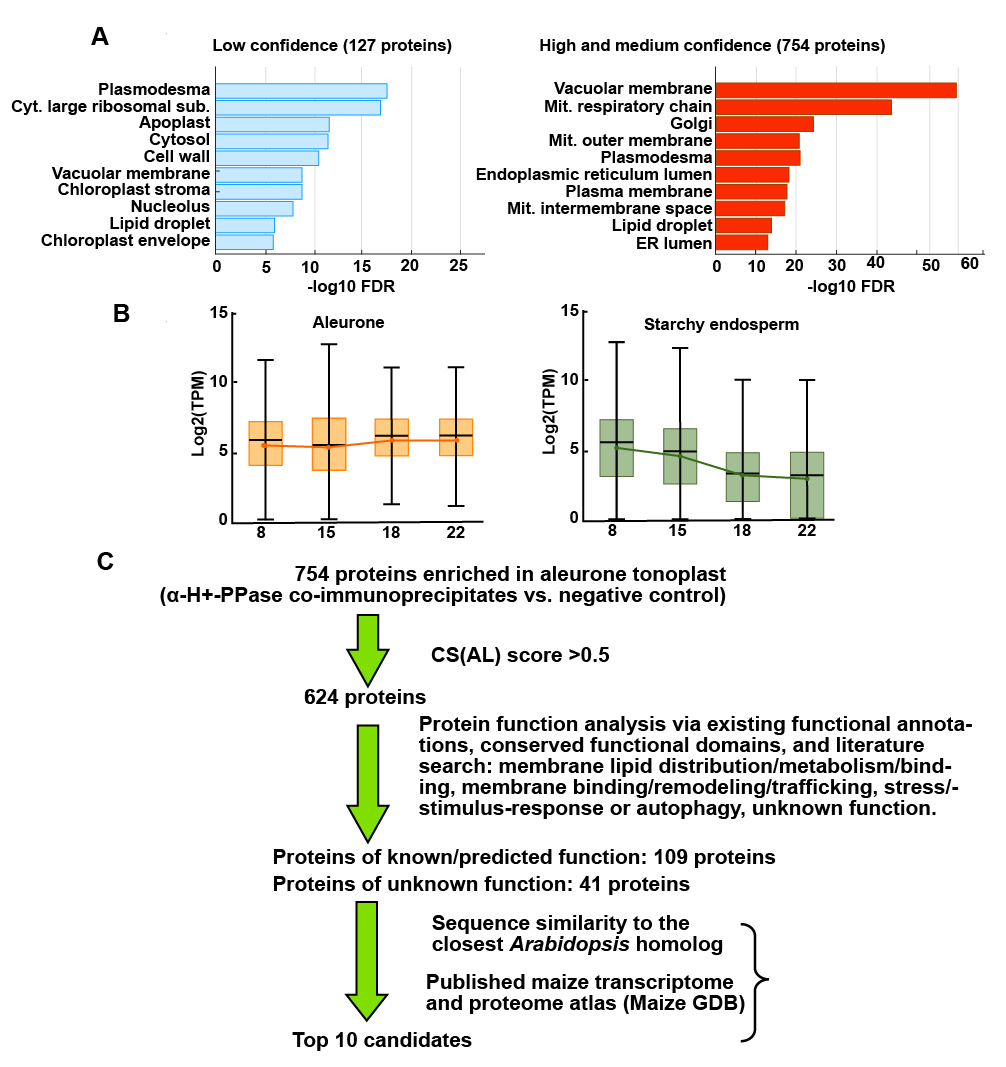
**

**Supplemental Figure S4.** Analysis and transcript abundance of proteins enriched in the anti-H+-PPase co-immunoprecipitates from the aleurone tonoplast fraction. (A) Top eight enriched GO terms of proteins detected at low confidence level in the immunoprecipitation with anti-H+-PPase antibodies. (B) Top eight enriched GO terms of proteins detected at medium and high confidence levels in the immunoprecipitation with anti-H+-PPase antibodies. (C) Steps in the selection of candidate proteins regulating microautophagy of storage proteins in maize aleurone cells. A total of 754 proteins were enriched with high- and medium- confidence in the co-immunoprecipitation with anti- H+-PPase antibodies. Based on their CS(AL) score higher than 0.5, we selected 624 proteins and based on their predicted functions in membrane binding/remodeling and in autophagy, the list was further reduced to 109 proteins. Of those, we selected 10 top candidates based on expression patterns and sequence similarity to relevant *Arabidopsis* proteins.

**
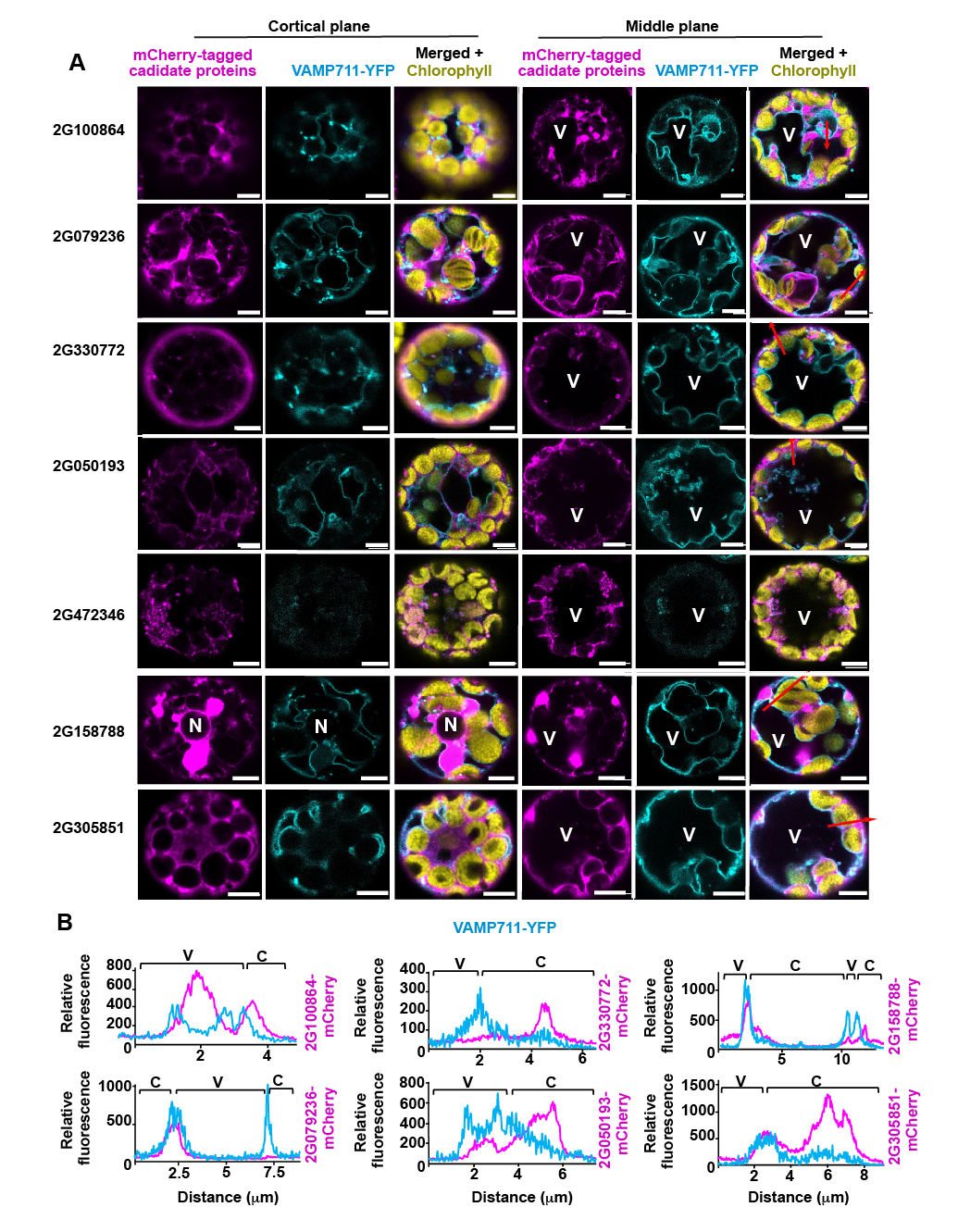
Supplemental Figure S5.** Expression of mCherry-tagged candidate proteins in Arabidopsis protoplasts expressing VAMP711-YFP. (A-H) Representative images of microautophagy candidate proteins (except for Rab7, PLDα5, and the EUL lectin protein) fused to mCherry and co-expressed with VAMP711-YFP. Overexpression of GRMZM2G472346 (BAG7)-mCherry induces cell death and only a few intact protoplasts expressing were imaged. (B) Fluorescence intensity profiles of mCherry-tagged proteins and VAMP711-YFP along the arrow depicted in protoplasts in (A). V, vacuole. Scale bars = 5 μm.

**
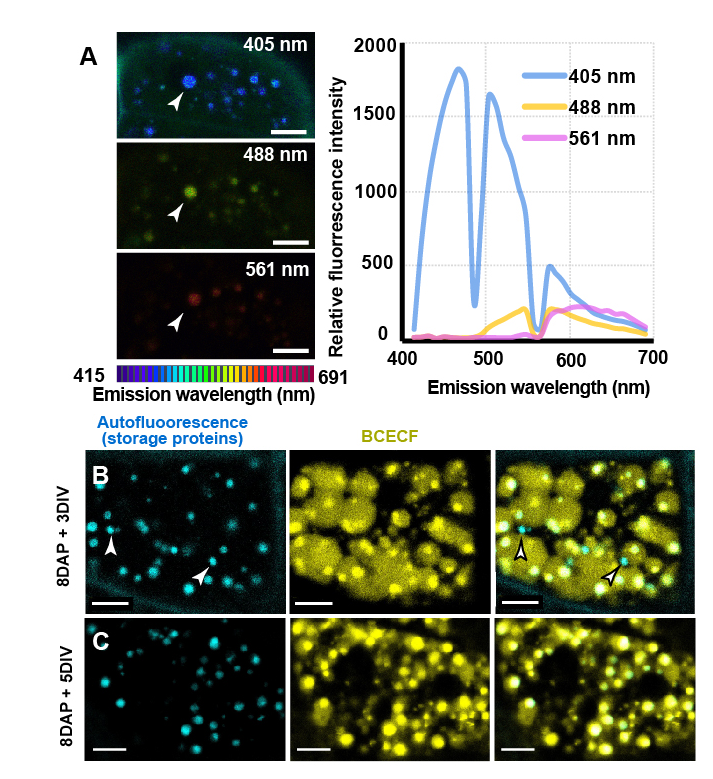
**

**Supplemental Figure S6.** Storage protein intrinsic fluorescence and changes in vacuolar morphology in aleurone cells during *in vitro* culture. (A) Intrinsic fluorescence emission of storage proteins excited at 405nm, 488nm, and 561nm in unstained aleurone cells. The three confocal micrographs on the left are from the same protein body (arrowhead) in an untreated aleurone cell excited with different wavelengths; the corresponding emission spectra are shown on the right. (B-C) Vacuoles in aleurone cells stained with BCECF-AM. Storage protein bodies are autofluorescent but become strongly stained by BCECF-AM once internalized into vacuoles by microautophagy. In aleurone cells of endosperms dissected at 8 DAP and kept in culture for 3 days (8 DAP + 3 DIV), vacuoles are relatively large (5-10 μm in maximum length) and irregular in shape. Storage protein bodies in the cytoplasm (not stained by BCECF-AM) can be seen at this stage. At 8DAP + 5 DIV, vacuoles become more spherical, smaller (1-3 μm); by this stage, all protein bodies are found inside protein storage vacuoles. Scale bars = 5 μm.

**
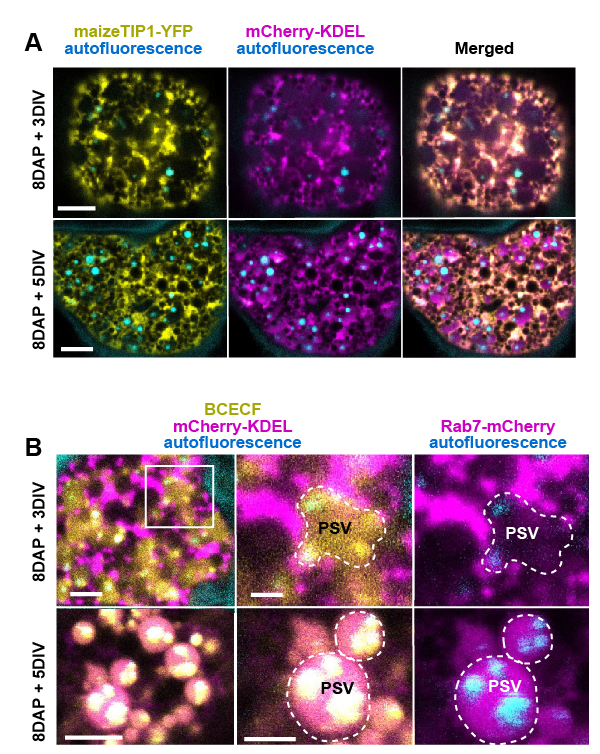
**

**Supplemental Figure S7.** Expression of fluorescently tagged proteins in *in vitro* grown endosperms by biolistic bombardment. (A) Co-expression of maize TIP1-YFP and mCherry-KDEL in aleurone cells from endosperms collected at 8 DAP and cultivated in vitro for either 3 days (8 DAP + 3DIV) or 5 days (8DAP+ 5DIV). At both stages, TIP1-YFP is mostly detected at the ER and does not reach the tonoplasts of the protein storage vacuoles. (B) Expression of mCherry-KDEL in aleurone cells. Whereas mCherry-KDEL is mostly detected in the ER at (8 DAP + 3DIV), 48 hours later (8DAP+ 5DIV), part of the signal comes from the lumen of protein storage vacuoles (PSVs). Scale bars = 5μm


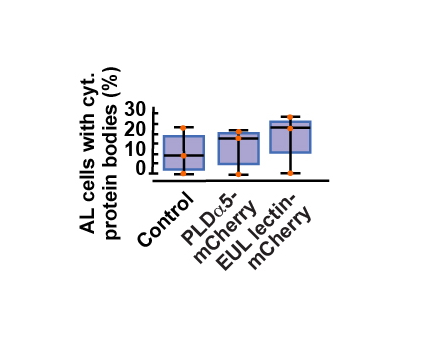


**Supplemental Figure S8.** Quantification of the percentage of aleurone cells in *in vitro* cultured endosperms with cytoplasmic protein bodies (outside the protein storage vacuoles) at 8 DAP + 3 DIV. The chart shows the mean of three trials, each with 5 to 14 cells analyzed. There is no statistical differences among the three samples based on a one-way ANOVA test.
